# ﻿Helminth and protozoan parasites of subterranean rodents (Chordata, Mammalia, Rodentia) of the world

**DOI:** 10.3897/zookeys.1151.97126

**Published:** 2023-03-01

**Authors:** Altangerel T. Dursahinhan, Daniel A. Kenkel, Scott L. Gardner

**Affiliations:** 1 Harold W. Manter Laboratory of Parasitology, W-529 Nebraska Hall, University of Nebraska State Museum, University of Nebraska-Lincoln, Lincoln, Nebraska, USA University of Nebraska State Museum Lincoln United States of America

**Keywords:** Bathyergidae, Cricetidae, Ctenomyidae, Endoparasite, Geomyidae, Heterocephalidae, Octodontidae, Spalacidae

## Abstract

Published studies and ten new unpublished records included herein reveal that approximately 174 species of endoparasites (helminths and protozoans) are known from 65 of 163 species of rodents that occupy the subterranean ecotope globally. Of those, 94 endoparasite species were originally described from these rodents. A total of 282 host-parasite associations are summarized from four major zoogeographic regions including Ethiopian, Palearctic/Oriental, Nearctic, and Neotropical. Thirty-four parasite records from the literature have been identified to only the level of the genus. In this summary, ten new records have been added, and the most current taxonomic status of each parasite species is noted. Interestingly, there are no data on endoparasites from more than 68% of described subterranean rodents, which indicates that discovery and documentation are at an early stage and must continue.

## ﻿Introduction

Subterranean rodents are animals adapted to live underground with minimal dependency on surface resources. They exhibit numerous adaptations to maintain their life activities in this niche including almost no externally visible neck, small eyes and ears, short legs, and very loose skin with soft fur that enables them to turn in their burrows with ease (Maser et al. 1981; Lacey et al. 2000). Conditions within the burrow systems are characterized by complete darkness, constant temperatures, relative humidity of 100% with low levels of air circulation, elevated carbon dioxide levels, and usually closed tunnels.

In contrast to mammals that live on the surface of the soil, subterranean rodents are completely acclimated to live in complex burrow systems below the surface. These animals have the capability to dig burrow systems through many types of friable soils (Lessa et al. 2008). The underground habitat has been invaded by rodents utilizing specialized digging methods in all zoogeographic regions of the world. However, rodents with the ability to dig tunnels underground by utilizing strong digging limbs as well as other morphological and physiological traits occur in all zoogeographic regions except Australia and Antarctica and adaptations by non-phylogenetically related groups to a subterranean existence are considered a product of convergent evolution (Losos 2011). Approximately 40% of the 6,500 mammal species of the world are rodents. Even though only 6.5% of all rodent species occupy the subterranean ecotope, these mammals play an essential part of the ecology in the areas in which they live, functioning as biological plows, cycling the earth, changing the landscape, promoting plant growth and ecological succession, and playing a critical role in cycling carbon and other nutrients through the soil. In the order Rodentia, a total of 163 extant subterranean species across 23 genera, within seven families, has been recognized with distributions throughout all continents except Antarctica and Australia (see Table 1) (Van Daele et al. 2007; Wilson et al. 2016, 2017).

**Table 1.** List of subterranean rodents. NA = Nearctic, Nt = Neotropical, E = Ethiopian, P = Palearctic, O = Oriental.

Suborder	Infraorder	Family	Subfamily	Tribe	#	Genus/Species	Region	
Castorimorpha	Geomorpha	Geomyidae	Geomyinae	Thomomyini	1	*Thomomysatrovarius* J. A. Allen, 1898	NA	NA
					2	*Thomomysbottae* (Eydoux & Gervais, 1836)	NA	NA
					3	*Thomomysbulbivorus* (Richardson, 1829)	NA	NA
					4	*Thomomysclusius* Coues, 1875	NA	NA
					5	*Thomomysidahoensis* Merriam, 1901	NA	NA
					6	*Thomomysmazama* Merriam, 1897	NA	NA
					7	*Thomomysmonticola* J. A. Allen, 1893	NA	NA
					8	*Thomomysnayarensis* Mathis et al., 2013	NA	NA
					9	*Thomomyssheldoni* Bailey, 1915	NA	NA
					10	*Thomomystalpoides* (Richardson, 1828)	NA	NA
					11	*Thomomystownsendii* (Bachman, 1839)	NA	NA
					12	*Thomomysumbrinus* (Richardson, 1829)	NA	NA
				Geomyini	13	*Geomysarenarius* Merriam, 1895	NA	NA
					14	*Geomysattwateri* Merriam, 1895	NA	NA
					15	*Geomysbreviceps* Baird, 1855	NA	NA
					16	*Geomysbursarius* (Shaw, 1800)	NA	NA
					17	*Geomysjugossicularis* Hooper, 1940	NA	NA
					18	*Geomysknoxjonesi* Baker & Genoways, 1975	NA	NA
					19	*Geomyslutescens* Merriam, 1890	NA	NA
					20	*Geomyspersonatus* True, 1889	NA	NA
					21	*Geomyspinetis* Rafinesque, 1817	Nt	Nt
					22	*Geomysstreckeri* Davis, 1943	NA	NA
					23	*Geomystexensis* Merriam, 1895	NA	NA
					24	*Geomystropicalis* Goldman, 1915	NA	NA
					25	*Zygogeomystrichopus* Merriam, 1895	Nt	Nt
					26	*Orthogeomysgrandis* (Thomas, 1893)	NA	Nt
Castorimorpha	Geomorpha	Geomyidae	Geomyinae	Geomyini	27	*Heterogeomyscavator* (Bangs, 1902)	Nt	Nt
					28	*Heterogeomyscherriei* (J. A. Allen, 1893)	Nt	Nt
					29	*Heterogeomysdariensis* (Goldman, 1912)	Nt	Nt
					30	*Heterogeomysheterodus* (Peters, 1865)	Nt	Nt
					31	*Heterogeomyshispidus* (Le Conte, 1852)	Nt	Nt
					32	*Heterogeomyslanius* Elliot, 1905	Nt	Nt
					33	*Heterogeomysunderwoodi* Osgood, 1931	Nt	Nt
					34	*Pappogeomysbulleri* (Thomas, 1892)	Nt	Nt
					35	*Cratogeomyscastanops* (Baird, 1852)	NA	Nt
					36	*Cratogeomysfulvescens* Merriam, 1895	NA	Nt
					37	*Cratogeomysfumosus* (Merriam, 1892)	Nt	Nt
					38	*Cratogeomysgoldmani* (Merriam, 1895)	NA	Nt
					39	*Cratogeomysmerriami* (Thomas, 1893)	Nt	Nt
					40	*Cratogeomysperotensis* Merriam, 1895	NA	Nt
					41	*Cratogeomysplaniceps* (Merriam, 1895)	NA	Nt
Hystricomorpha	Histricognathi	Ctenomyidae			42	*Ctenomysandersoni* Gardner et al., 2014	Nt	Nt
					43	*Ctenomysargentinus* J. R. Contreras & Berry, 1982	Nt	Nt
					44	*Ctenomysaustralis* Rusconi, 1934	Nt	Nt
					45	*Ctenomysazarae* Thomas, 1903	Nt	Nt
					46	*Ctenomysbergi* Thomas, 1902	Nt	Nt
					47	*Ctenomysbicolor* Miranda-Ribeiro, 1914	Nt	Nt
					48	*Ctenomysboliviensis* Waterhouse, 1848	Nt	Nt
					49	*Ctenomysbonettoi* J. R. Contreras & Berry, 1982	Nt	Nt
					50	*Ctenomysbrasiliensis* de Blainville, 1826	Nt	Nt
					51	*Ctenomyscolburni* J. A. Allen, 1903	Nt	Nt
					52	*Ctenomyscoludo* Thomas, 1920	Nt	Nt
					52	*Ctenomyscoludo* Thomas, 1920	Nt	Nt
					53	*Ctenomysconoveri* Osgood, 1946	Nt	Nt
Hystricomorpha	Histricognathi	Ctenomyidae			54	*Ctenomyscoyhaiquensis* Kelt & Gallardo, 1994	Nt	Nt
					55	*Ctenomysdorbignyi* Contreras & Contreras, 1984	Nt	Nt
					56	*Ctenomysdorsalis* Thomas, 1900	Nt	Nt
					57	*Ctenomysemilianus* Thomas & S. Leger, 1926	Nt	Nt
					58	*Ctenomyserikacuellarae* Gardner et al., 2014	Nt	Nt
					59	*Ctenomysfamosus* Thomas, 1920	Nt	Nt
					60	*Ctenomysflamarioni* Travi, 1981	Nt	Nt
					61	*Ctenomysfodax* Thomas, 1910	Nt	Nt
					62	*Ctenomysfochi* Thomas, 1919	Nt	Nt
					63	*Ctenomysfrater* Thomas, 1902	Nt	Nt
					64	*Ctenomysfulvus* Philippi, 1860	Nt	Nt
					65	*Ctenomysgoodfellowi* Thomas, 1921	Nt	Nt
					66	*Ctenomyshaigi* Thomas, 1919	Nt	Nt
					67	*Ctenomysibicuiensis* Freitas et al., 2012	Nt	Nt
					68	*Ctenomysjohannis* Thomas, 1921	Nt	Nt
					69	*Ctenomysjuris* Thomas, 1920	Nt	Nt
					70	*Ctenomysknighti* Thomas, 1919	Nt	Nt
					71	*Ctenomyslami* Freitas, 2001	Nt	Nt
					72	*Ctenomyslatro* Thomas, 1918	Nt	Nt
					73	*Ctenomyslessai* Gardner et al., 2014	Nt	Nt
					74	*Ctenomysleucodon* Waterhouse, 1848	Nt	Nt
					75	*Ctenomyslewisi* Thomas, 1926	Nt	Nt
					76	*Ctenomysmagellanicus* Bennett, 1836	Nt	Nt
					77	*Ctenomysmariafarelli* Azurduy, 2005	Nt	Nt
					78	*Ctenomysmaulinus* Philippi, 1872	Nt	Nt
					79	*Ctenomysmendocinus* Philippi, 1869	Nt	Nt
					80	*Ctenomysminitus* Nehring, 1887	Nt	Nt
					81	*Ctenomysnattereri* Wagner, 1848	Nt	Nt
					82	*Ctenomysoccultus* Thomas, 1920	Nt	Nt
					83	*Ctenomysopimus* Wagner, 1848	Nt	Nt
					84	*Ctenomysosvaldoreigi* J. R. Contreras, 1985	Nt	Nt
					85	*Ctenomysparaguayensis* J. R. Contreras, 2000	Nt	Nt
					86	*Ctenomyspearsoni* Lessa & Langguth, 1983	Nt	Nt
					87	*Ctenomysperrensi* Thomas, 1896	Nt	Nt
					88	*Ctenomysperuanus* Sanborn & Pearson, 1947	Nt	Nt
					89	*Ctenomyspilarensis* J. R. Contreras, 1993	Nt	Nt
					90	*Ctenomyspontifex* Thomas, 1918	Nt	Nt
					91	*Ctenomysporteousi* Thomas, 1916	Nt	Nt
					92	*Ctenomyspundti* Nehring, 1900	Nt	Nt
					93	*Ctenomysrionegrensis* Langguth & Abella, 1970	Nt	Nt
					94	*Ctenomysroigi* J. R. Contreras, 1988	Nt	Nt
					95	*Ctenomysrondoni* Miranda-Ribeiro, 1914	Nt	Nt
					96	*Ctenomysrosendopascuali* J. R. Contreras, 1995	Nt	Nt
					97	*Ctenomystalarum* Thomas, 1898	Nt	Nt
					98	*Ctenomystorquatus* Lichtenstein, 1830	Nt	Nt
					99	*Ctenomystuconax* Thomas, 1925	Nt	Nt
					100	*Ctenomystucumanus* Thomas, 1900	Nt	Nt
					101	*Ctenomystulduco* Thomas, 1921	Nt	Nt
					102	*Ctenomyssaltarius* Thomas, 1912	Nt	Nt
					103	*Ctenomysscagliai* J. R. Contreras, 1999	Nt	Nt
					104	*Ctenomyssericeus* J. A. Allen, 1903	Nt	Nt
					105	*Ctenomyssociabilis* Pearson & Christie, 1985	Nt	Nt
					106	*Ctenomyssteinbachi* Thomas, 1907	Nt	Nt
					107	*Ctenomysvalidus* J. R. Contreras et al., 1977	Nt	Nt
					108	*Ctenomysviperinus* Thomas, 1926	Nt	Nt
					109	*Ctenomysyatesi* Gardner et al., 2014	Nt	Nt
					110	*Ctenomysyolandae* J. R. Contreras & Berry, 1984	Nt	Nt
Hystricomorpha	Histricognathi	Octodontidae			111	*Spalacopuscyanus* (Molina, 1782)	Nt	Nt
		Heterocephalidae			112	*Heterocephalusglaber* Rüppell, 1842	E	E
		Bathyergidae			113	*Heliophobiusargenteocinereus* Peters, 1846	E	E
					114	*Bathyergusjanetta* Thomas & Schwann, 1904	E	E
					115	*Bathyergussuillus* (Schreber, 1782)	E	E
					116	*Georychuscapensis* (Pallas, 1778)	E	E
					117	*Cryptomyshottentotus* (Lesson, 1826)	E	E
					118	*Fukomysamatus* (Wroughton, 1907)	E	E
					119	*Fukomysanselli* (Burda et al., 1999)	E	E
					120	*Fukomysbocagei* (de Winton, 1897)	E	E
					121	*Fukomysdamarensis* (Ogilby, 1838)	E	E
					122	*Fukomysdarlingi* (Thomas 1895)	E	E
					123	*Fukomysfoxi* (Thomas, 1911)	E	E
					124	*Fukomyskafuensis* (Burda et al., 1999)	E	E
					125	*Fukomysmechowii* (Peters, 1881)	E	E
					126	*Fukomysochraceocinereus* (Heuglin, 1846)	E	E
					127	*Fukomysvandewoestijneae* Van Daele et al., 2013	E	E
					128	*Fukomyswhytei* (Thomas, 1897)	E	E
					129	*Fukomyszechi* (Matschie, 1900)	E	E
Myomorpha		Cricetidae	Arvicolinae	Prometheomyini	130	*Prometheomysschaposchnikowi* Satunin, 1901	P	P
				Ellobiusini	131	*Ellobiusalaicus* Vorontsov et al., 1969	P	P
					132	*Ellobiusfuscocapillus* (Blyth, 1843)	P	P
					133	*Ellobiuslutescens* Thomas, 1897	P	P
					134	*Ellobiustalpinus* (Pallas, 1770)	P	P
					135	*Ellobiustancrei* Blasius, 1884	P	P
		Spalacidae	Myospalacinae		136	*Myospalaxarmandii* (Milne-Edwards, 1867)	P	P
					137	*Myospalaxaspalax* (Pallas, 1776)	P	P
					138	*Myospalaxepsilanus* Thomas, 1912	P	P
					139	*Myospalaxmyospalax* (Laxmann, 1773)	P	P
					140	*Myospalaxpsilurus* (Milne-Edwards, 1874)	P	P
					141	*Eospalaxbaileyi* (Thomas, 1911)	P	P
					142	*Eospalaxcansus* (Lyon, 1907)	P	P
					143	*Eospalaxfontanierii* (Milne-Edwards, 1867)	P	P
					144	*Eospalaxrothschildi* (Thomas, 1911)	P	P
					145	*Eospalaxrufescens* (J. A. Allen, 1909)	P	P
					146	*Eospalaxsmithii* (Thomas, 1911)	P	P
			Rhizomyinae	Rhizomyini	147	*Rhizomyspruinosus* (Blyth, 1851)	P	O
					148	*Rhizomyssinensis* Gray, 1831	P	O
					149	*Rhizomyssumatrensis* (Raffles, 1821)	O	O
					150	*Cannomysbodius* (Hodgson, 1841)	O	O
				Tachyoryctini	151	*Tachyoryctesmacrocephalus* (Rüppell, 1842)	E	E
					152	*Tachyoryctessplendens* (Rüppell, 1835)	E	E
			Spalacinae		153	*Spalaxantiquus* Méhely, 1909	P	P
					154	*Spalaxarenarius* Reshetnik, 1939	P	P
					155	*Spalaxgiganteus* Nehring, 1898	P	P
					156	*Spalaxgraecus* Nehring, 1898	P	P
					157	*Spalaxistricus* Méhely, 1909	P	P
					158	*Spalaxmicrophthalmus* Güldenstädt, 1770	P	P
					159	*Spalaxuralensis* Tiflov & Usov, 1939	P	P
					160	*Spalaxzemni* (Erxleben, 1777)	P	P
					161	*Nannospalaxehrenbergi* Nehring, 1898	P	P
					162	*Nannospalaxleucodon* (Nordmann, 1840)	P	P
					163	*Nannospalaxxanthodon* (Nordmann, 1840)	P	P

Based on macroevolutionary patterns derived from the study of the fossil record, subterranean rodent species diversity has appeared to have oscillated since early Oligocene time [ca. 36 million years ago, (mya)]. The Geomyidae Bonaparte, 1845 and the Bathyergidae Waterhouse, 1841 have the greatest diversity among all subterranean rodent families relative to the number of genera found throughout evolutionary time and identified thus far as fossil taxa (Cook et al. 2000). Fluctuation cycles in diversification, known as taxon pulses (Erwin 1985) appear to have been driven by local, regional, and global climate oscillations, and explained by the Stockholm Paradigm, which seeks to provide an understanding of the evolution of host-parasite/pathogen systems via the evolutionary process of species diversification following mass extinctions (Brooks et al. 2019).

### ﻿Ethiopian subterranean rodents

Subterranean rodents in the Ethiopian zoogeographic region are represented by twenty species in seven genera across three families (Heterocephalidae, Bathyergidae, and Spalacidae) including *Heterocephalus* Rüppell, 1842, *Heliophobius* Peters, 1846, *Bathyergus* Illiger, 1811, *Georychus* Illiger, 1811, *Cryptomys* Gray, 1864, *Fukomys* Kock et al., 2006, and *Tachyoryctes* Rüppell, 1835 (see Landry 1957; Patterson and Upham 2014; Wilson et al. 2016).

### ﻿Nearctic subterranean rodents

Species of the family Geomyidae are endemic to the Nearctic and northern Neotropics and are known collectively as pocket gophers due to presence of fur-lined cheek pouches in all species. They are a monophyletic group of subterranean rodents that share common ancestry with rodents of the family Heteromyidae (Wilson et al. 2016). Pocket gophers inhabit a wide geographic range, extending from a northernmost limit in southwest and south-central Canada through the central and western United States, southeast into central Florida, and south into Mexico and through Central America into Panama and near the Rio Atrato in northern Colombia (Hall 1981; Alberico 1990; Solari et al. 2013). As in most subterranean rodents, pocket gophers are fusiform in shape, having a naked and sensitive tail (they can run backwards as fast as they can forwards, using their tail as a rear-guide sensor (Gardner, pers. obs.). They have small pinnae, loose skin, and their fur-lined cheek pouches are used only for food transport (Howard and Childs 1959; Maser et al. 1981; Hafner 1982; Honeycutt and Williams 1982; Hafner et al. 1994). The family consists of seven extant genera and 41 species (Wilson et al. 2016). The genus *Thomomys* Wied-Niewied, 1839, has 12 species and many subspecies, making this genus the most speciose of the family Geomyidae (see Patton 2005).

### ﻿Palearctic subterranean rodents

Thirty-two species of subterranean rodents of seven genera in two families, including Cricetidae Fischer, 1817, and Spalacidae Gray, 1821, occur in the Palearctic region. Those include *Prometheomys* Satunin, 1901, and *Ellobius* Fischer, 1814, in the family Cricetidae, which includes the subfamily Arvicolinae Gray, 1821. The genera *Myospalax* Laxmann, 1769, *Eospalax* Allen, 1938, *Rhizomys* Gray, 1831, *Cannomys* Thomas, 1915, *Spalax* Guldenstaedt, 1770, and *Nannospalax* (Nordmann, 1840) are in the family Spalacidae which includes the subfamilies Myospalacinae Lilljeborg, 1866, Rhizomyinae Winge, 1887, and Spalacinae Gray, 1821. Among all subterranean forms of the Rodentia, those occurring in the Palearctic region have the most extensive geographic distribution. Based on fossil evidence, the first known subterranean species of rodents appeared in the early Pliocene of Asia (Repenning 1984; Repenning et al. 1990). *Rhizomyspruinosus* (Blyth, 1851) and *Rhizomyssinensis* Gray, 1831 occur in the Palearctic and Oriental zoogeographic regions. At the current time, only two species of subterranean rodents are known from the Oriental region, and those include *Rhizomyssumatrensis* (Raffles, 1821) and *Cannomysbodius* (Hodgson, 1841).

### ﻿Neotropical subterranean rodents

The Neotropical subterranean rodents are represented by two hystricognath Caviomorph families, the Ctenomyidae Lesson, 1842 and Octodontidae Waterhouse, 1839. The family Ctenomyidae currently includes only species in the genus *Ctenomys* Blainville, 1826 which are known as the tuco-tucos, with approximately 69 described species. These rodents occur in suitable habitats with a geographic distribution from southern Peru and southwestern Brazil south to Tierra de Fuego through Chile, Argentina, Bolivia, Paraguay, and Uruguay (Reig et al. 1990; Gardner et al. 2014). The fossil record indicates that species that can be allocated to the family Ctenomyidae originated ca. 10 mya (Cook et al. 2000), with rapid diversification in the genus *Ctenomys* commencing at ca. 3 mya (Parada et al. 2011). Interestingly, the single subterranean species in the Octodontidae which are the sister taxon of the Ctenomyidae includes the monotypic *Spalacopuscyanus* (Molina, 1782) and these occur only in central Chile.

### ﻿Endoparasites

Our analysis shows that endoparasites have been found and reported from fewer than 40% of known species of subterranean rodents world-wide. There are several factors that could potentially explain this lack of reported data as researchers face several challenges when trapping subterranean rodents; without prior training, just finding and then determining active subterranean mammal burrow mounds is difficult. There could be thousands of burrow mounds, but researchers need acute field expertise to identify freshly dug burrows to capture these animals. Moreover, setting subterranean rodent traps is labor intensive and time-consuming, demanding lots of patience, physical strength, and luck.

Another problem is that sampling of species of subterranean rodents has not been systematically carried out and most collecting was done over time that was rather scattered and sporadic, and very few collections included parasites in their investigations. Many previous studies have failed to record comprehensive data during their collections of mammals and other vertebrates, discarding the internal organs of collected mammals without further examination. This practice resulted in significant gaps in parasite data with black holes regarding their faunas of both ecto- and endoparasites. Parasites discovered in research projects contain vital information about themselves and their host life history, consisting of information that we cannot ignore. The work presented here represents a synthesis of all available literature on the endoparasites of subterranean rodents of the world, as such, some references and works may have been missed, but we hope that this list provides a starting point for other researchers interested in this area of study.

## ﻿Materials and methods

The current checklist was created by accumulating all published references arranged in a chronologically ordered tabular form representing globally each continent. The taxonomic status of each host and parasite species are up to date and represent the most current classifications. Most of the early literature was located in the reprint library of the H.W. Manter Laboratory of Parasitology in the University of Nebraska State Museum. Some of the literature was obtained from the Digital Commons at University of Nebraska-Lincoln Libraries while several rare international references were obtained through interlibrary loan. For new records presented herein, some samples were collected during the field Parasitology class in western Nebraska and others were included from field work by S.L. Gardner in the 1980’s the 1990’s and earlier. Except for a few instances that we detail in the results, we used the original taxonomic names of both the hosts and parasites as published in the original literature. Throughout this paper, we used the zoogeographic terminology first established by Wallace (1876) (Rueda et al. 2013).

## ﻿Results

### ﻿Literature review

**Ethiopian subterranean rodent endoparasites**

**Figure 1.**
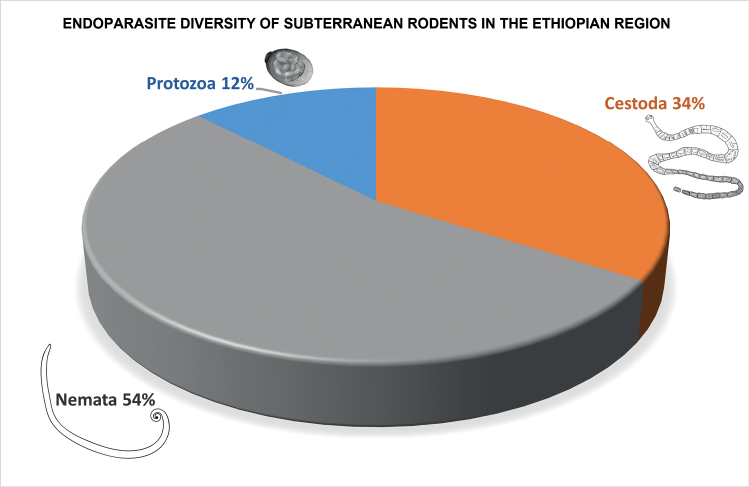
Pie diagram representing percentage taxon composition of the higher classification of endoparasite diversity found infecting subterranean rodents from the Ethiopian zoogeographic region derived from records in the literature published from 1864 through 2018. The Nemata are the most speciose representing 54% of the total endoparasite fauna, followed by Cestoda (34%), and Protozoa (12%).

See graphical summary in Fig. 1 and endoparasite diversity list in Table 2.

**Table 2.** Endoparasite species diversity of Ethiopian subterranean rodents and their known original hosts. Authorities are given for parasite and host species.

Host species	Parasite species	References
*Bathyergussuillus* (Schreber, 1782)	*Mammalakismacrospiculum* (Ortlepp, 1939)	Lutermann et al. 2012
	*Ortleppstrongylusbathyergi* Ortlepp, 1939	De Graaff 1964
	*Paralibyostrongylusbathyergi* (Ortlepp, 1939)	Lutermann et al. 2012
	*Rodentolepis* Spasskii, 1954	Lutermann et al. 2012
	*Taenia* Linnaeus, 1758	Lutermann et al. 2012
	*Trichostrongylus* Looss, 1905	De Graaff 1964
	*Trichuris* Roederer, 1761	Lutermann et al. 2012
*Cryptomyshottentotus* (Lesson, 1826)	*Ascaropsafricana* (Sandground, 1933)	Lutermann et al. 2013
	*Heligmonina* Baylis, 1928	Viljoen et al. 2011
	*Inermicapsifermadagascariensis* (Davaine, 1870)	De Graaff 1981
	*Mammalakismacrospiculum* (Ortlepp, 1939)	Archer et al. 2017
	*Mathevotaenia* Akhumyan, 1946	Viljoen et al. 2011
	*Neoheligmonella* Durette-Desset, 1971	Archer et al. 2017
	*Protospirura* Seurat, 1914	Viljoen et al. 2011
	*Raillietina* Fuhrman, 1920	Lutermann et al. 2013
	*Trichuris* Roederer, 1761	Archer et al. 2017
*Fukomysanselli* (Burda et al., 1999)	*Hexametra* Travassos, 1920	Lutermann et al. 2018
	*Inermicapsifer* Janicki, 1910	Lutermann et al. 2018
	*Mammalakiszambiensis* Junker et al., 2017	Junker et al. 2017
	*Protospiruramuricola* (Gedoelst, 1916)	Lutermann et al. 2018
	*Protospiruranumidica* Seurat, 1914	Lutermann et al. 2018
	*Protospirura* Seurat, 1914	Lutermann et al. 2018
	Rodentolepiscf.microstoma (Dujardin, 1945)	Lutermann et al. 2018
*Fukomyskafuensis* (Burda et al., 1999)	*Inermicapsifermadagascariensis* (Davaine, 1870)	Scharff et al. 1997
	*Protospiruramuricola* (Gedoelst, 1916)	Scharff et al. 1997
*Fukomysmechowii* (Peters, 1881)	*Capillaria* Zeder, 1800	Scharff et al. 1997
	*Inermicapsifermadagascariensis* (Davaine, 1870)	Scharff et al. 1997
	*Protospiruramuricola* (Gedoelst, 1916)	Scharff et al. 1997
	*Raillietina* Fuhrman, 1920	Scharff et al. 1997
*Georychuscapensis* (Pallas, 1778)	*Coenurusspalacis* Diesing, 1864	Diesing 1864
	*Echinococcus* Rudolphi, 1801	De Graaff 1964; Hüttner and Romig 2009
	*Trichuris* Roederer, 1761	Lutermann et al. 2012
*Heliophobiusargenteocinereus* Peters, 1846	*Eimeriaburdai* Koudela et al., 2000	Koudela et al. 2000
	*Eimeriaheliophobii* Modrý et al., 2005	Modrý et al. 2005
	*Eimerianafuko* Modrý et al., 2005	Modrý et al. 2005
	*Eimeriayamikamiae* Modrý et al., 2005	Modrý et al. 2005
	*Inermicapsiferarvicanthidis* (Kofend, 1917)	Baruš et al. 2003; Tenora et al. 2003
	*Protospiruramuricola* (Gedoelst, 1916)	Baruš et al. 2003; Tenora et al. 2003
*Heterocephalusglaber* Rüppell, 1842	*Eimeriaheterocephali* Levine & Ivens, 1965	Levine and Ivens 1965
*Tachyoryctessplendens* (Rüppell, 1835)	*Taeniabrauni* Setti, 1897	Fain 1956
*Tachyoryctesmacrocephalus* (Rüppell, 1842)	*Ascaropsafricana* (Sandground, 1933)	Schmidt and Canaris 1968

Diesing (1864) reported the first helminth parasite species from a subterranean rodent host, where *Taeniaspalacis* (Diesing, 1864) was reported from *Georychuscapensis* (Pallas, 1779) collected from Port Natal, South Africa.

Ortlepp (1939) described three new nematode species from the Cape dune mole-rat, *Bathyergussuillus* (Schreber, 1782) (Bathyergidae: Rodentia) collected from Strandfontein and Cape Town, South Africa including: *Libyostrongylusbathyergi* Ortlepp, 1939, *Longistriatabathyergi*, and *Mammalakismacrospiculum* (see Ortlepp 1939; Inglis 1991). These represent the first known reports of parasitic nematodes from African subterranean rodents. Interestingly, all these species have been reclassified into different genera and are currently known as *Paralibyostrongylusbathyergi*, *Ortleppstrongylusbathyergi*, and *Mammalakismacrospiculum*, (see De Graaff 1964; Lutermann and Bennett 2012).

Fain (1956) reported *Taeniabrauni* Setti, 1897, from *Tachyoryctessplendens* (Rüppell, 1835) collected in Ruanda-Urundi, East Africa. After a period of several years, De Graaff (1964) mentioned that in a personal communication that he had with Ortlepp an unidentified tapeworm was obtained by Ortlepp from *Bathyergussuillus*, collected at Houtbay, near Cape Town. Also, De Graaff (1964) wrote that Ortlepp told him that he found cysts of an unidentified species of *Echinococcus* Rudolphi, 1801 obtained from the muscles of the abdominal cavity as well as liver of *G.capensis* collected at Wynberg, near Cape Town, South Africa (Hüttner and Romig 2009).

Levine and Ivens (1965) described the first coccidian parasite, *Eimeriaheterocephali* from the mucosal epithelial cells of the cecum of a *Heterocephalusglaber* specimen collected at Somaliland or Kenya, South Africa.

Schmidt and Canaris (1968) reported *Ascaropsafricana* (Sandground, 1933) from *Tachyoryctesmacrocephalus* (Rüppell, 1842) collected from Njoro, Kenya, East Africa.

Several years later, De Graaff (1981) reported *Inermicapsifermadagascariensis* (Davaine, 1870) from the Common mole-rat, *Cryptomyshottentotus* (Lesson, 1826) (Bathyergidae: Hystricomorpha), collected from Shingwedzi, South Africa.

Scharff et al. (1997) reported *Inermicapsifermadagascariensis* from the small intestine, and *Protospiruramuricola* (Gedoelst, 1916) from the colon of *Fukomyskafuensis* (Burda et al., 1999) collected from Itezhi-Tezhi, Zambia. They also found an unidentified species of *Calodium* Moravec, 1982 (syn. *Capillaria* Zeder, 1800) (eggs only), *I.madagascariensis*, and an unknown *Raillietina* Fuhrman, 1920, from the small intestine, and *P.muricola* from the abdominal cavity of *Fukomysmechowii* (Peters, 1881) collected from Ndole, Zambia. The discovery of *P.muricola* in the abdominal cavity was probably a result of these nematodes moving from the stomach during or after the necropsy event of the individual *F.mechowii* mentioned.

Koudela et al. (2000) described *Eimeriaburdai*, as a new species of coccidian from the subterranean African silvery mole-rat, *Heliophobiusargenteocinereus* Peters, 1846, collected from Lubalashi Province, central Zambia.

Baruš et al. (2003) studied the relative concentration of heavy metals in helminth parasites; several Silvery mole-rats, *H.argenteocinereus*, were necropsied for their internal parasite tissues collected from the Blantyre-Limbe region of Malawi, southeastern Africa. As a result, two species of helminths were found, including *Inermicapsiferarvicanthidis* (Kofend, 1917) and *Protospiruramuricola*, and these specimens were later examined for four heavy metal elements (cadmium, copper, lead, and zinc), and compared against the liver and muscle tissues of their hosts. The same species of parasites from these same hosts were reported by Tenora et al. (2003).

Modrý et al. (2005) described three new species of *Eimeria* from the Silvery mole-rat *H.argenteocinereus* from Malawi, including: *Eimeriaheliophobii*, *E.nafuko*, and *E.yamikamiae* extracted from the fecal samples from the host specimens.

Viljoen et al. (2011), in an ecological study of the role of host traits, season, and group size on parasite burdens in a cooperative breeding mammal, captured 87 individual mole-rats were from the Tshwane region of South Africa in different seasons. Three helminths that were not identified to the species level were obtained from the small intestine of *Cryptomyshottentotus*, including *Heligmonina* sp. Baylis, 1928, *Mathevotaenia* sp. Akhumyan, 1946, and *Protospirura* sp. Seurat, 1914.

Lutermann and Bennett (2012), during a year-long joint research and eradication project for *Bathyergussuillus* at Cape Town International Airport, Cape Town, South Africa, found these rodents infected with three species of nematodes, including: *Mammalakismacrospiculum*, *Paralibyostrongylusbathyergi*, and *Trichuris* sp. Roederer, 1761, and two species of tapeworms, *Rodentolepis* sp. Spasskii, 1954, and *Taenia* sp. Linnaeus, 1758.

Just one year later, Lutermann et al. (2013), during the study on energetic beneﬁts and costs of parasitism in a cooperative mammal identified *Raillietina* sp., and *Ascaropsafricana* from the small intestine of *Cryptomyshottentotus* collected from KwaZulu-Natal, South Africa.

Archer et al. (2017), in a seasonal comparative study between two Common mole-rat populations found *Mammalakismacrospiculum*, *Neoheligmonella* Durette-Desset, 1971, and *Trichuris* sp. in *Cryptomyshottentotus* collected from two different habitats, including an arid site, 25 km outside of Kamieskroon, the Northern Cape and a mesic site near Darling, western Cape, South Africa.

Junker et al. (2017) described a new species of ascaridid nematode, *Mammalakiszambiensis* acquired from the large intestine and cecum of Ansell’s mole-rat, *Fukomysanselli* (Burda et al., 1999), captured from west of Lusaka at Mukulaikwa Farm Block, Zambia.

Lutermann et al. (2018) reported the following gastrointestinal parasites from Ansell’s mole-rat, *F.anselli* in Zambia. Those include *Hexametra* sp. Travassos, 1920, *Inermicapsifer* sp. Janicki, 1910, *Protospiruramuricola*, *Protospiruranumidica* Seurat, 1914, and Rodentolepiscf.microstoma (Dujardin, 1945).

#### ﻿Palearctic subterranean rodent endoparasites

**Figure 2.**
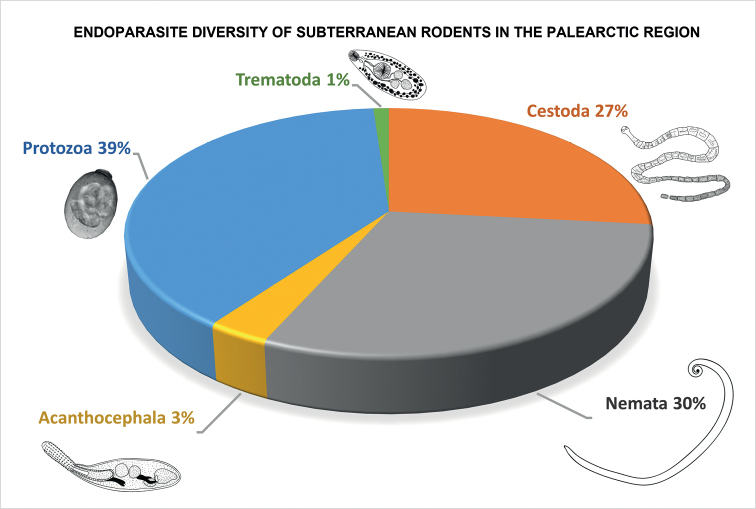
Pie chart showing percentage of infection summary of the higher-level classification of endoparasite diversity among Palearctic subterranean rodents derived from a survey of published records from 1927 through 2022. Protozoa constitute the greatest diversity of endoparasites accounting for 39% of the total parasite species recovered followed by Nemata (30%), Cestoda (27%), Acanthocephala (3%), and the Trematoda coming in at only 1%.

See graphical summary in Fig. 2 and endoparasite list in Table 3.

**Table 3.** Endoparasite species diversity of Palearctic subterranean rodents and their known original hosts. Authorities are given for parasite and host species.

Host species	Parasite species	References
*Cannomysbodius* (Hodgson, 1841)	*Hymenolepisdiminuta* (Rudolphi, 1819)	Malsawmtluangi and Tandon 2009
*Ellobiusfuscocapillus* (Blyth, 1843)	*Syphaciaobvelata* (Rudolphi, 1802)	Arzamani et al. 2017
*Ellobiuslutescens* Thomas, 1897	*Eimerialutescenae* Musaev & Veisov, 1963	Musaev and Veisov 1965a
*Ellobiustalpinus* (Pallas, 1770)	*Aprostatandryamacrocephala* Douthitt, 1915	Tokobaev 1960
	*Catenotaeniapusilla* Goeze, 1782	Zanina and Tokobaev 1962a
	*Echinococcusmultilocularis* Leuckart, 1863	Tokobaev 1960
	*Eimeriaellobii* Svanbaev, 1965	Musaev and Veisov 1965a
	*Eimeriakazakhstanensis* Levine, 1965	Levine and Ivens 1965
	*Eimeriatadshikistanica* Veisov, 1964	Musaev and Veisov 1965a
	*Eimeriatalpini* Levine, 1965	Levine and Ivens 1965
	*Hymenolepisdiminuta* (Rudolphi, 1819)	Zanina and Tokobaev 1962a
	*Mesocestoides* Vaillant, 1863	Tokobaev 1960
	*Moniliformismoniliformis* Bremser, 1811	Zanina and Tokobaev 1962a
	*Nomadolepisellobii* Makarikov et al., 2010	Makarikov et al. 2010
	*Physocephalusellobii* Schulz, 1927	Schultz 1927
	*Hydatigera* (syn. *Taenia*) *taeniaeformis* (Batsch, 1786)	Zanina and Tokobaev 1962a
*Ellobiustancrei* Blasius, 1884	*Arostrilepisbatsaikhani* Dursahinhan et al., 2022	Dursahinhan et al. 2022
	*Echinococcusmultilocularis* Leuckart, 1863	Afonso et al. 2015
*Eospalaxbaileyi* (Thomas, 1911)	*Eimeriabaileyii* Cao et al., 2014	Cao et al. 2014
	*Eimeriafani* Cao et al., 2014	Cao et al. 2014
	*Eimeriamenyuanensis* Cao et al., 2014	Cao et al. 2014
	*Eimeriamyospalacensis* Cao et al., 2014	Cao et al. 2014
	*Ransomusqinghaiensis* Ming et al., 2004	Ming et al. 2004
	*Versteria* (syn. *Taenia*) *mustelae* Gmelin, 1790	Zhao, et al. 2014
*Eospalaxfontanierii* (Milne-Edwards, 1867)	*Echinococcusmultilocularis* Leuckart, 1863	Craig 2006
	*Heligmopteragiraudouxi* Elias et al., 2002	Elias, et al. 2002
	*Heligmopteraquerei* Elias et al., 2002	Elias, et al. 2002
*Myospalaxmyospalax* (Laxmann, 1773)	*Echinococcusmultilocularis* Leuckart, 1863	Shaykenov and Mahmutov 1968
	*Heligmopterasibirica* Shakhmatova, 1990	Shakhmatova 1990
	*Heligmosomummyospalaxi* Nadtochii, 1970	Nadtochii 1970
	*Hymenolepisrymzhanovi* Makarikov & Tkach, 2013	Makarikov and Tkach 2013
	*Moniliformisclarki* (Ward, 1917)	Vlasenko and Krivopalov 2017
	*Paranoplocephala* Lühe, 1910	Vlasenko and Krivopalov 2017
	*Versteriamustelae* (Gmelin, 1790)	Vlasenko and Krivopalov 2017
*Myospalaxpsilurus* (Milne-Edwards, 1874)	*Ascaropsstrongylina* (Rudolphi, 1819)	Ganzorig et al. 1999
*Nannospalaxehrenbergi* Nehring, 1898	*Eimeriaadiyamanensis* Sayin, 1980	Sayın 1980
	*Eimeriaanzanensis* Couch et al, 1993	Couch et al. 1993
	*Eimeriacarmelensis* Couch et al, 1993	Couch et al. 1993
	*Eimeriacelebii* Sayin, 1980	Sayın 1980
	*Eimeriaharanica* Sayin, 1980	Sayın 1980
	*Eimeriamarasensis* Sayin, 1980	Sayın 1980
	*Eimeriamicrospalacis* Golemansky & Darawish, 1992	Golemansky and Darwish 1992
	*Eimeriaoytuni* Sayin, 1980	Sayın 1980
	*Eimeriaspalacensis* Couch et al, 1993	Couch et al. 1993
	*Eimeriatorosicum* Sayin, 1980	Sayın 1980
	*Eimeriaurfensis* Sayin, 1980	Sayın 1980
	*Ganguleterakisspalaxi* Kozlov & Yangolenko, 1963	Wertheim and Nevo 1971
	*Gongylonemalongispiculum* Schulz, 1927	Wertheim and Nevo 1971
	*Heligmonella* Mönnig, 1927	Wertheim and Nevo 1971
	*Heligmoninanevoi* Wertheim & Nevo, 1971	Wertheim and Nevo 1971
	*Isosporaspalacensis* Couch et al, 1993	Couch et al. 1993
	*Microcephaloidesnevoi* (Fair et al., 1990) Haukisalmi 2009	Fair et al. 1990; Haukisalmi 2009
	*Trichurismuris* (Schrank, 1788)	Wertheim and Nevo 1971
*Nannospalaxleucodon* (Nordmann, 1840)	*Aprostatandrya* Kirshenblat, 1938	Andreiko 1963a
	*Ascarisspalacis* Shults & Aloyan, 1950	Shults and Aloyan 1950
	*Coenurusparviuncinatus* Kirschenblatt, 1939	Korniushin and Sharpilo 1986
	*Eimeriacelebii* Sayin, 1980	Nalbantoğlu et al. 2010
	*Eimeriaelliptica* Sayin et al., 1977	Sayin et al. 1977
	*Eimerialalahanensis* Sayin, et al., 1977	Sayin et al. 1977
	*Eimerialeucodonica* Veisov, 1975	Veisov 1975
	*Eimeriamaralikiensis* Veisov, 1975	Veisov 1975
	*Eimeriaoytuni* Sayin, 1980	Nalbantoğlu et al. 2010
	*Eimeriaspalacis* Sayin et al., 1977	Sayin et al. 1977
	*Eimeriatalikiensis* Veisov, 1975	Veisov 1975
	*Eimeriatorosicum* Sayin, 1980	Nalbantoğlu et al. 2010
	*Eimeriaturkmenica* Sayin et al., 1977	Sayin et al. 1977
	*Eimeriatuzdili* Sayin, et al., 1977	Sayin et al. 1977
	*Heligmosomumspalacis* Kirsenblat, 1965	Mészáros 1968
	*Heligmosomummoldovensis* Andreiko, 1963	Andreiko 1963a
	*Isosporaanatolicum* Sayin, et al., 1977	Sayin et al. 1977
	*Longistriataspalacis* Sharpilo, 1973	Sharpilo 1973a
	*Mammalakisspalacis* Marcu, 1930	Andreiko 1963a
	*Moniliformismoniliformis* Bremser, 1811	Murai 1968
	*Taeniastraminea* (Goeze, 1782) Spasskii 1954	Andreiko 1963a
*Prometheomysschaposchnikowi* Satunin, 1901	*Dicrocoeliumdendriticum* (Rudolphi, 1819)	Razumova 1957
	*Heligmosomumhalli* (Schulz, 1926)	Razumova 1957
	*Microcephaloides* Haukisalmi et al., 2008	Razumova 1957
	*Taeniapolyacantha* Leuckart, 1856	Razumova 1957
	*Hydatigera* (syn. *Taenia*) *taeniaeformis* (Batsch, 1786)	Razumova 1957
*Rhizomyspruinosus* (Blyth, 1851)	*Mammalakisspumosa* (Schneider, 1866)	Chaisiri et al. 2017
*Rhizomyssinensis* Gray, 1831	*Cryptosporidiumoccultus* Kváč, 2018	Wei et al. 2019
	*Cryptosporidiumparvum* Tyzzer, 1912	Wei et al. 2019
*Spalaxarenarius* Reshetnik, 1939	*Longistriataspalacis* Sharpilo, 1973	Sharpilo 1973a
*Spalaxgraecus* Nehring, 1898	*Heligmosomumspalacis* Kirsenblat, 1965	Kirshenblat 1965a
*Spalaxmicrophthalmus* Güldenstädt, 1770	*Ganguleterakisspalaxi* Kozlov & Yangolenko, 1963	Kozlov and Yangolenko 1963a
	*Gongylonemalongispiculumspalacis* Schulz, 1927	Schultz 1927
	*Longistriataspalacis* Sharpilo, 1973	Sharpilo 1973a
	*Mammalakisspalacis* Marcu, 1930	Marcu 1930
	*Hydatigera* (syn. *Taenia*) *taeniaeformis* (Batsch, 1786)	Sharpilo 1976
	*Trichurisspalacis* (Petrov & Potechina, 1953)	Petrov and Potechina 1953

Interestingly, even though helminthology began in Europe (the western Palearctic) in the late 1800’s with the work of Leuckart, it was not until the 1920’s when Schulz (1927) described the first two species of helminth parasites from two species of subterranean rodents from the Palearctic region. First, *Physocephalusellobii* Schulz, 1927 was found from the stomach of *Ellobiustancrei* Blasius, 1884 collected from Kotlyrevsky, the northern Caucasus region of Russia. Second, *Gongylonemalongispiculumspalacis* Schulz, 1927 was described as the first subspecies found under the mucous membrane of the stomach of *Spalaxmicrophthalmus* Güldenstädt, 1770 collected from the village Kurichya Kosa near the Don River region north of the coast of the Sea of Azov, Russia. Soon after, Marcu (1930) described *Mammalakisspalacis* Marcu, 1930 also obtained from *S.microphthalmus* collected from Romania.

Somewhat later, Schulz and Aloyan (1950), described *Ascarisspalacis* Schulz & Aloyan, 1950 from Lesser mole-rat, *Nannospalaxleucodon* (Nordmann, 1840). Kirshenblat collected the materials included in the description from near the small towns of Amamla and Chandura, of the Spitakskii and Akhalkalakskii regions of Armenia, in 1947. All these nematode samples were found from the small intestines of the hosts, necropsied by Aloyan in 1948.

Petrov and Potechina (1953) described *Trichurisspalacis* from *S.microphthalmus* collected from an unspecified locality in Ukraine.

Razumova (1957) reported the following helminth parasites found in specimens of the Long-clawed mole vole, *Prometheomysschaposchnikowi* Satunin, 1901, captured from Ossetia, Russia. These include *Dicrocoeliumdendriticum* (Rudolphi, 1819), *Heligmosomumhalli* (Schulz, 1926), *Microcephaloides* Haukisalmi et al., 2008, *Taeniapolyacantha* Leuckart, 1856, and *Hydatigera* (syn. *Taenia*) *taeniaeformis* (Batsch, 1786).

Tokobaev (1960) reported the collection of *Ellobiustalpinus* (Pallas, 1770) from the Kyrgyz Republic and found larvae of *Echinococcusmultilocularis* from the liver. In the same report, he reported *Aprostatandryamacrocephala* Douthitt, 1915, from the small intestine and larvae of *Mesocestoides* Vaillant, 1863 from the body cavity, liver, and small intestines. In work on mole voles just a short time later, Zanina and Tokobaev (1962) reported *Catenotaeniapusilla* Goeze, 1782, *Hymenolepisdiminuta* Rudolphi, 1819, *Moniliformismoniliformis* Bremser, 1811, and *Hydatigera* (syn. *Taenia*) *taeniaeformis* (Batsch, 1786) from *E.talpinus* collected in Tajikistan.

Andreiko (1963) reported that from 1959 through 1962, 70 Lesser mole-rats, (*Nannospalaxleucodon*) collected from the central part of Moldova, Romania had the following helminths: *Mammalakisspalacis* from the cecum, *Taeniastraminea* (Goeze, 1782) Spasskii, 1954 and unidentified species of *Aprostatandrya* Kirshenblat, 1938 from the small intestine. In addition, she described *Heligmosomummoldovensis* Andreiko, 1963 from the small intestine of *N.leucodon*.

Kozlov and Yangolenko (1963) described *Ganguleterakisspalaxi* Kozlov & Yangolenko, 1963 from *Spalaxmicrophthalmus* collected from Ukraine.

Kirshenblat (1965) described a new species of nematode *Heligmosomumspalacis* from the small intestine of the mole-rat *Spalaxgraecus* Nehring, 1898 collected from Chernivtsi, Ukraine.

Levine and Ivens (1965) described two species of *Eimeria* Fischer, 1814 from the Northern mole vole, including: *Ellobiuskazakhstanensis* Levine & Ivens, 1965, and *Ellobiustalpini* Levine & Ivens, 1965 from the fecal of *Ellobiustalpinus* collected from Kazakhstan.

Musaev and Veisov (1963) described *Eimerialutescenae* Musaev & Veisov, 1963 from *Ellobiuslutescens* Thomas, 1897 from Nakhichevanskaia, Azerbaijan. In addition, two *Eimeria* (Schneider, 1875) species were reported with their descriptions, including: *Eimeriaellobii* Svanbaev, 1965 and *Eimeriatadshikistanica* Veisov, 1964 from *Ellobiustalpinus* collected from Tajikistan.

Shaykenov and Mahmutov (1968) reported *Echinococcusmultilocularis* found in *Myospalaxmyospalax* (Laxmann, 1773) collected from eastern Kazakhstan. This record is considered a new intermediate host for this tapeworm. Also in the same year, Mészáros (1968) reported the occurrence of *Heligmosomumspalacis* recovered from the Lesser mole-rat, *Nannospalaxleucodon*, collected from Hungary.

Murai (1968) recorded the Lesser mole-rat, *N.leucodon*, as a new host of *Moniliformismoniliformis*. The acanthocephalid parasite was extracted from the small intestines of two individuals of Lesser mole-rats. Also, *Heligmosomumspalacis* was found in the host. The study has conducted near Hajdubagos village, Hajdu-Bihar, in Hungary.

Nadtochii (1970), during a study of helminth parasites of rodents in far eastern Russia, the author described *Heligmosomummyospalaxi* Nadtochii, 1970 obtained from the small intestine of *Myospalaxmyospalax* collected from the seashore of eastern Russia.

Wertheim and Nevo (1971), during a study of helminths of birds and mammals from Israel recovered several species of helminth parasites from the Middle East blind mole-rat, *Nannospalaxehrenbergi* Nehring, 1898 including *Ganguleterakisspalaxi*, *Gongylonemalongispiculum* Schulz, 1927, *Trichurismuris* (Schrank, 1788), and one unidentified nematode in the genus *Heligmonella* Mönnig, 1927. They also described *Heligmoninanevoi* Wertheim & Nevo, 1971 from the same host species.

Sharpilo (1973) described *Longistriataspalacis* from the small intestine of Lesser mole-rat, *Nannospalaxleucodon*. He reported that this nematode species was also found from *Spalaxarenarius* Reshetnik, 1939, and *Spalaxmicrophthalmus*. These specimens were all collected from Ukraine.

Sharpilo (1976), during a study of helminth parasites of rodent fauna in Ukraine, reported *Hydatigera* (syn. *Taenia*) *taeniaeformis* from *Spalaxmicrophthalmus*.

Veisov (1975) described three new species of coccidia of the genus *Eimeria* Schneider, 1875 from *Nannospalaxleucodon*, including *Eimeriamaralikiensis* Veisov, 1975 and *Eimeriatalikiensis* Veisov, 1975 collected from Talnisk and Maralik Aniisk regions, Armenian, also, describing *Eimerialeucodonica* Veisov, 1975 from the Talinsk region only.

Sayin et al. (1977), during a survey of Lesser mole-rats, *Nannospalaxleucodon*, in Lalahan district in Ankara, Turkey, described six new species of coccidia in the genus *Eimeria* Schneider, 1875 including *E.elliptica*Sayin et al., 1977, *E.lalahanensis*Sayin et al., 1977, *E.spalacis*Sayin et al., 1977, *E.turkmenica*Sayin et al., 1977, *E.tuzdili*Sayin et al., 1977, and *Isosporaanatolicum*Sayin et al., 1977.

Sayın (1980), during a survey conducted from 1976 through 1978, studied 41 individuals of the Middle East blind mole-rats, *Nannospalaxehrenbergi*, from Urfa, Adiyaman, and Maras provinces in Turkey. As a result, seven new species of coccidia of the genus *Eimeria* Schneider, 1875 were described. Those include *E.adiyamanensis* Sayın, 1980, *E.celebii* Sayın, 1980, *E.haranica* Sayın, 1980, *E.marasensis* Sayın, 1980, *E.oytuni* Sayın, 1980, *E.torosicum* Sayın, 1980, and *E.urfensis* Sayın, 1980.

Korniushin and Sharpilo (1986) reported a larval *Taenia* which they reported as *Coenurusparviuncinatus* Kirschenblatt, 1939 obtained from *Nannospalaxleucodon* collected from Armenia.

Fair et al. (1990) described a new species of tapeworm, *Microcephaloidesnevoi*Fair et al., 1990 from the Middle East blind mole-rat *Nannospalaxehrenbergi* in Masada, Golan Heights, Israel. This species has been redescribed by Haukisalmi (2009).

Shakhmatova (1990) described *Heligmopterasibirica* Shakhmatova, 1990 found from the Siberian zokor, *Myospalaxmyospalax*, collected from the Gorno-Altai autonomous region of Russia.

Golemansky and Darwish (1992) described *Eimeriamicrospalacis* Golemansky & Darwish, 1992 from the Middle East blind mole-rat, *Nannospalaxehrenbergi*, collected from the regions of Damascus and Latakia, western Syria.

Couch et al. (1993) described four coccidian parasites obtained from the Middle East blind mole-rat, *Nannospalaxehrenbergi*, collected from 12 different localities in Israel including *Eimeriaanzanensis*Couch et al., 1993, *E.carmelensis*Couch et al., 1993, *E.spalacensis*Couch et al., 1993, and *Isosporaspalacensis*Couch et al., 1993.

Ganzorig et al. (1999) redescribed *Ascaropsstrongylina* (Rudolphi, 1819) from the Transbaikal zokor, *Myospalaxpsilurus* (Milne-Edwards, 1874) collected from near the Halh Gol River, Dornod province, eastern Mongolia.

Elias et al. (2002), during a joint program of French, British, and China on echinococcosis screening in Zhang County, China (Gansu), two new species of *Heligmoptera* Nadtochiy, 1977 were described from the small intestines of the Chinese zokor, *Eospalaxfontanierii* (Milne-Edwards, 1867) including: *Heligmopteragiraudouxi* Elias & Durette-Desset, 2002, and *Heligmopteraquerei* Elias & Durette-Desset, 2002 with the new description of the genus.

More recently in China, Ming et al. (2004) described *Ransomusqinghaiensis*Ming et al., 2004 from the cecum of the Plateau zokor, *Eospalaxbaileyi* (Thomas, 1911) collected from Qilian County, Qinghai province.

Craig (2006), in a survey and epidemiological assessment of human alveolar echinococcosis in 33 provinces of China, listed the Chinese zokor, *Eospalaxfontanierii* as one of the intermediate hosts of *Echinococcusmultilocularis*.

Malsawmtluangi and Tandon (2009) reported *Hymenolepisdiminuta* attained from the Lesser bamboo rat, *Cannomysbodius* (Hodgson, 1841) collected from Mizoram, northeast India.

Nalbantoğlu et al. (2010) reported three species of coccidia acquired from the feces of the Lesser mole-rat, *Nannospalaxleucodon*, collected from the Eryaman district of Ankara, Turkey. Those are *Eimeriacelebii* , *E.oytuni* Sayin, 1980, and *E.torosicum* Sayin, 1980. In the same year, Makarikov et al. (2010) described the cestode *Nomadolepisellobii*Makarikov et al., 2010, simultaneously establishing a new genus for the tapeworm that was obtained from the small intestine of the Northern mole vole, *Ellobiustalpinus*, collected from southwestern Siberia, Russia.

Soon after, Makarikov and Tkach (2013) described *Hymenolepisrymzhanovi* Makarikov & Tkach, 2013 from the small intestine of the Siberian zokor, *Myospalaxmyospalax* collected from eastern Kazakhstan.

Cao et al. (2014) described four new species of *Eimeria* from the Plateau zokor, *Eospalaxbaileyi*, collected from Haibei area, Qinghai Province, China. The parasites include *Eimeriabaileyii*Cao et al., 2014, *Eimeriafani*Cao et al., 2014, *Eimeriamenyuanensis*Cao et al., 2014, and *Eimeriamyospalacensis*Cao et al., 2014. In the same year, Zhao et al. (2014) identified *Versteria* (syn. *Taenia*) *mustelae* (Gmelin, 1790) using DNA sequencing of larval cysts found in the Plateau zokor, *Eospalaxbaileyi* collected from Datong County, east of Qinghai province, China. In this study, no data were provided on number of individuals infected.

Afonso et al. (2015) reported *Echinococcusmultilocularis* from the livers of Eastern mole voles, *Ellobiustancrei* which acts as the intermediate host for this cestode, collected from Sary Mogol, Alay valley, Kyrgyzstan. The authors also noted that the definitive hosts were local domestic dogs, whose feces were examined for *E.multilocularis*. The parasite samples from the dogs were genetically identical to those found in the intermediate host.

In 2017, a flurry of activity resulted from workers in the field. Vlasenko and Krivopalov (2017) reported *Moniliformisclarki* (Ward, 1917), *Paranoplocephala* Lühe, 1910 and larvae of *Versteriamustelae* (Gmelin, 1790) from *Myospalaxmyospalax* collected from the southern Tomsk region, Russia. Then, Arzamani et al. (2017) reported *Syphaciaobvelata* (Rudolphi, 1802) (probably a misidentification as *S.obvelata* occurs only in species of *Mus*) obtained in the Southern mole vole, *Ellobiusfuscocapillus* (Blyth, 1843), collected from north Khorasan province of northeast Iran. Finally in 2017, Chaisiri et al. (2017), during an ecological study of host-parasite associations, reported *Mammalakisspumosa* (Schneider, 1866) from *Rhizomyspruinosus* in Cambodia.

Wei et al. (2019) reported *Cryptosporidiumparvum* Tyzzer, 1912 and *C.occultus* Kváč, 2018 found in the Chinese bamboo rat, *Rhizomyssinensis*, collected from south-central China.

Dursahinhan et al. (2022) described *Arostrilepisbatsaikhani* from the Zaisan mole vole, *Ellobiustancrei* collected from Baitag Bogd, Hovd province, western Mongolia.

#### ﻿Endoparasites of Nearctic and northern Neotropical subterranean rodents

**Figure 3.**
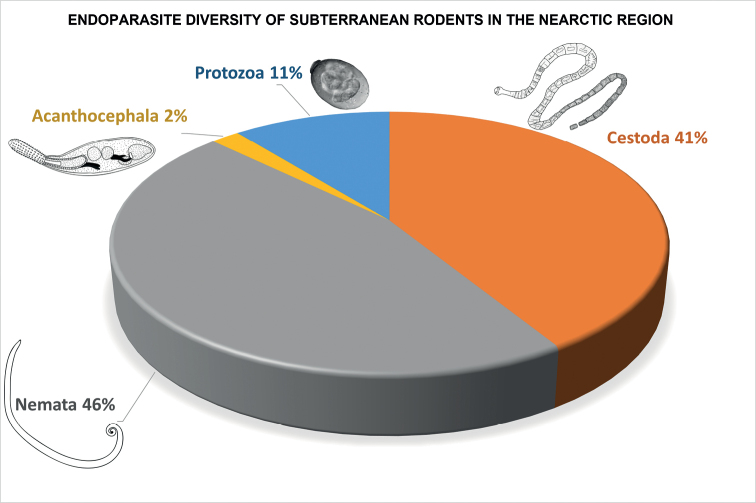
Percentage taxon composition pie diagram of the higher classification of endoparasite diversity occurring in Nearctic subterranean rodents (Family Geomyidae) derived from literature records published from 1857 through 2020. Among these endoparasites, the Nemata represent 46% of the species found followed by Cestoda (41%), Protozoa (11%), and Acanthocephala at just 2%.

See graphical summary in Fig. 3 and endoparasite list Table 4.

**Table 4.** Endoparasite species diversity of Nearctic and Neotropical regions of subterranean rodents in the family Geomyidae and their known hosts. Authorities are given for parasite and host species. The new host-parasite associations recorded in this work are denoted by ‘Present study’ in bold.

Host species	Parasite species	References
*Cratogeomyscastanops* (Baird, 1852)	*Calodiumamericanum* (Read, 1949)	**Present study**
	*Eimeriageomydis* Skidmore, 1929	**Present study**
	*Monoecocestus* sp. Beddard, 1914	**Present study**
	*Vexillataconvoluta* Caballero & Cerecero, 1943	**Present study**
*Cratogeomysmerriami* (Thomas, 1893)	*Paraspidoderauncinata* Travassos, 1914	Lamothe-Argumedo et al. 1997
	*Vexillataconvoluta* Caballero & Cerecero, 1943	Caballero and Cerecero 1943
*Cratogeomysplaniceps* (Merriam, 1895)	*Hymenolepiscratogeomyos* Gardner et al., 2020	Gardner et al. 2020
*Geomysattwateri* Merriam, 1895	*Monoecocestuscentroovarium* Dronen et al., 1994	Dronen et al. 1994
	*Protospiruraascaroidea* Hall, 1916	LeBrasseur 2017
	*Vexillatageomyos* Falcón-Ordaz et al., 2006	Falcón-Ordaz et al. 2006
*Geomysbreviceps* Baird, 1855	*Eimeriageomydis* Skidmore, 1929	Upton et al. 1992
	*Litomosoideswesti* Gardner & Schmidt, 1986	Pitts et al. 2000
	*Monoecocestusanoplocephaloides* (Douthitt, 1915)	Douthitt 1915
	*Protospiruraascaroidea* Hall, 1916	Hall 1916; English 1932
*Geomysbursarius* (Shaw, 1800)	*Andryamacrocephala* Douthitt, 1915	Douthitt 1915; Hansen 1950; Ubelaker and Downhower 1965; Bartel and Gardner 2000
	*Anoplocephaloidesinfrequens* (Douthitt, 1915)	Douthitt 1915; Ubelaker and Downhower 1965; Bartel and Gardner 2000; Rausch 1976
	*Anoplocephaloidesvariabilis* (Douthitt, 1915)	Douthitt 1915; Rausch 1976
	*Calodiumamericanum* (Read, 1949)	Bartel and Gardner 2000
	*Calodiumhepaticum* (Bancroft, 1893)	Ubelaker and Downhower 1965
	*Cittotaeniaperplexa* Stiles, 1897	Burnham 1953
	*Eimeriageomydis* Skidmore, 1929	Skidmore 1929; Levine and Ivens 1965
	*Hymenolepisdiminuta* (Rudolphi, 1819)	Burnham 1953
	*Hymenolepisgeomydis* Gardner & Schmidt, 1988	Gardner and Schmidt 1988
	*Hymenolepisweldensis* Gardner & Schmidt, 1988	Gardner and Schmidt 1988; Bartel and Gardner 2000; Haukisalmi et al. 2010
	*Litomosafilaria* (Beneden, 1873)	Burnham 1953
	*Litomosoideswesti* Gardner & Schmidt, 1986	Gardner and Schmidt 1986
	*Moniliformisclarki* (Ward, 1917)	Bartel and Gardner 2000
	*Monocercomonoides* Travis, 1932	Rissky 1962
	*Monoecocestusanoplocephaloides* (Douthitt, 1915)	Burnham 1953
	*Oochoristica* Lűhe, 1898	Douthitt 1915
	*Ostertagia* Ransom, 1907	Burnham 1953
	*Paranoplocephalainfrequens* (Douthitt, 1915)	Ubelaker and Downhower 1965
	*Physalopteralimbata* Leidy, 1856	Bartel and Gardner 2000
	*Protospiruraascaroidea* Hall, 1916	English 1932; LeBrasseur 2017
	*Protospiruramurisascaroides* (Hall, 1916)	Burnham 1953
	*Pseudocittotaeniapraecoquis* (Stiles, 1985)	Stiles 1895
	*Ransomusrodentorum* Hall, 1916	Bartel and Gardner 2000
*Geomysjugossicularis* Hooper, 1940	*Anoplocephaloidesvariabilis* (Douthitt, 1915)	**Present study**
*Geomyslutescens* Merriam, 1890	*Hymenolepisweldensis* Gardner & Schmidt, 1988	Gardner et al. 2020
	*Litomosoideswesti* Gardner & Schmidt, 1986	**Present study**
	*Physalopteralimbata* Leidy, 1856	**Present study**
	*Ransomusrodentorum* Hall, 1916	**Present study**
	*Monoecocestusanoplocephaloides* (Douthitt, 1915)	Burnham 1953
*Geomyspersonatus* True, 1889	*Litomosoideswesti* Gardner & Schmidt, 1986	Pitts et al. 2000
	*Protospiruraascaroidea* Hall, 1916	LeBrasseur 2017
*Geomyspinetis* Rafinesque, 1817	*Mastophorusmurisascaroides* (Gmelin, 1790)	Hubbell and Goff 1939
*Geomystexensis* Merriam, 1895	*Eimeriageomydis* Skidmore, 1929	Upton et al. 1992
	*Hymenolepis* Weinland, 1858	LeBrasseur 2017
	*Protospiruraascaroidea* Hall, 1916	LeBrasseur 2017
*Heterogeomysheterodus* (Peter, 1865)	*Hobergiairazuensis* Gardner et al., 2020	Gardner et al. 2020
*Orthogeomysgrandis* (Thomas, 1893)	*Eimeriaorthogeomys* Lainson, 1968	Lainson 1968
*Thomomysbottae* (Eydoux & Gervais, 1836)	*Arostrilepishorrida* (von Linstow, 1901)	Schiller 1952; Voge 1955; Gardner 1985
	*Catenotaeniadendritica* (Goeze, 1782)	Voge 1955
	*Catenotaenialinsdalei* McIntosh, 1941	McIntosh 1941
	*Eimeriathomomysis* Levine et al., 1957	Levine et al. 1957; Levine and Ivens 1965
	*Heligmosomoidesthomomyos* Gardner & Jasmer, 1983	Gardner and Jasmer 1983
	*Hymenolepiscitelli* (McLeod, 1933)	Voge 1955; Jasmer 1980
	*Litomosoidesthomomydis* Gardner, 1986	Gardner and Schmidt 1986
	*Monocercomonoides* Travis, 1932	Gardner and Jasmer 1983
	*Monoecocestusanoplocephaloides* (Douthitt, 1915)	Hansen 1950
	*Ransomusrodentorum* Hall, 1916	Jasmer 1980
	*Trichurisfossor* Hall, 1916	Jasmer 1980; Douglas 1969
*Thomomysbulbivorus* (Richardson, 1829)	*Arostrilepishorrida* (von Linstow, 1901)	Gardner 1985
	*Arostrilepisschilleri* Makarikov et al., 2012	Makarikov et al. 2012
	*Heligmosomoidesthomomyos* Gardner & Jasmer, 1983	Gardner 1985; Gardner and Jasmer 1983
	*Hymenolepistualatinensis* Gardner, 1985	Gardner 1985
	*Ransomusrodentorum* Hall, 1916	Gardner 1985
	*Trichurisfossor* Hall, 1916	Gardner 1985
*Thomomysclusius* Coues, 1875	*Ransomusrodentorum* Hall, 1916	**Present study**
	*Trichurisfossor* Hall, 1916	**Present study**
*Thomomysmonticola* J. A. Allen, 1893	*Arostrilepishorrida* (von Linstow, 1901)	Howard and Childs 1959
	*Trichuris* Roederer, 1761	Ingles 1952
*Thomomystalpoides* (Richardson, 1828)	*Andryamacrocephala* Douthitt, 1915	Rausch and Schiller 1949
	*Anoplocephaloidesinfrequens* (Douthitt, 1915)	Frandsen and Grundmann 1961; Todd et al. 1971
	*Anoplocephaloidesvariabilis* (Douthitt, 1915)	Rausch 1976; Frandsen and Grundmann 1961; Todd et al. 1971; Lubinsky 1957
	*Arostrilepishorrida* (von Linstow, 1901)	Grundmann, et al. 1976; Frandsen and Grundmann 1961
	*Ascarislaevis* Leidy, 1856	Grundmann et al. 1976; Frandsen and Grundmann 1961
	*Calodiumhepaticum* (Bancroft, 1893)	Ubelaker and Downhower 1965; Lubinsky 1957; Dikmans 1932; Tryon 1947; Lubinsky 1956; Rausch 1961; Tryon and Cunningham 1968
	*Catenotaenialinsdalei* McIntosh, 1941	Todd et al. 1971
	*Eimeriafitzgeraldi* Todd & Tryon, 1970	Todd et al. 1971; Todd and Tryon 1970
	*Eimeriajemezi* Wilber et al., 1994	Wilber et al. 1994
	*Eimeriathomomysis* Levine et al., 1957	Levine and Ivens 1965; Levine et al. 1957
	*Hymenandryathomomyis* Smith, 1954	Smith 1954
	*Hymenolepiscitelli* (McLeod, 1933)	Frandsen and Grundmann 1961
	*Hymenolepisdiminuta* (Rudolphi, 1819)	Rankin 1945
	*Litomosoidescarinii* (Travassos, 1919)	Lubinsky 1957
	*Litomosoidesthomomydis* Gardner, 1986	Gardner and Schmidt 1986
	*Nippostrongylusmuris* (Yokogawa, 1920)	Frandsen and Grundmann 1961
	*Protospiruraascaroidea* Hall, 1916	Todd et al. 1971
	*Pseudocittotaeniaglandularis* Beveridge, 1978	Beveridge 1978
	*Pseudocittotaeniapraecoquis* (Stiles, 1985)	Grundmann et al. 1976; Frandsen and Grundmann 1961; Smith 1951
	*Ransomusrodentorum* Hall, 1916	Grundmann et al. 1976; Frandsen and Grundmann 1961
	*Trichurisfossor* Hall, 1916	Hall 1916; Grundmann et al. 1976; Frandsen and Grundmann 1961; Lubinsky 1957; Todd and Lepp 1972
	*Versteriamustelae* (Gmelin, 1790)	Lubinsky 1957
	*Vexillatavexillata* (Hall, 1916)	Todd et al. 1971
*Thomomysumbrinus* (Richardson, 1829)	*Arostrilepishorrida* (von Linstow, 1901)	Frandsen and Grundmann 1961
	*Ascarislaevis* Leidy, 1856	Frandsen and Grundmann 1961
	*Hymenolepiscitelli* (McLeod, 1933)	Frandsen and Grundmann 1961
	*Moniliformisclarki* (Ward, 1917)	Frandsen and Grundmann 1961
	*Paruterinacandelabraria* (Goeze, 1782)	Frandsen and Grundmann 1961
	*Ransomusrodentorum* Hall, 1916	Frandsen and Grundmann 1961
	*Trichurisfossor* Hall, 1916	Frandsen and Grundmann 1961

Leidy in (1857), at a meeting of the Academy of Natural Sciences of Philadelphia, displayed some warbles taken from an evidently incapacitated pocket gopher by the side of the road, identified as *T.borealis* [probably a synonym of *T.talpoides*] near the Bridger’s pass summit of the Rocky Mountains. This record represents the first known report of an endoparasite from a member of the rodent family Geomyidae. Soon after the groundbreaking work by Leidy, Charles Wardell Stiles (1895) reported the first helminth parasite from a geomyid when he described *Pseudocittotaeniapraecoquis* (Stiles, 1895) from *Geomysbursarius* (Shaw, 1800) collected near Ames, Iowa (Stiles, 1897).

Hall (1912) reported on the parasite fauna of Colorado and recorded several nematodes and some unidentified cestodes from *Thomomysfossor* J.A. Allen (probably a syn. of *T.talpoides*). Soon after, Herman Douthitt (1915) described four new species of anoplocephalid cestodes from pocket gophers collected from the central United States. *Anoplocephaloidesvariabilis* (Douthitt, 1915), *A.infrequens* (Douthitt, 1915), and *Andryamacrocephala* Douthitt, 1915 were all described from specimens taken from *G.bursarius* collected from Illinois, Minnesota, and North Dakota. *Monoecocestusanoplocephaloides* (Douthitt, 1915) was described from some specimens taken from *Geomysbreviceps* Baird collected near Norman, Oklahoma. Douthitt (1915) also reported one unidentified species of *Oöchoristica* Luhe, 1898, and one immature form of *Cittotaenia*, now known as *Pseudocittotaenia*, Tenora, 1976 from *G.bursarius*. Douthitt (1915) also reported numerous individuals of eight different species of *Hymenolepis* from two species of pocket gophers including: *G.bursarius* collected in Illinois, Wisconsin, Minnesota, North Dakota, and Manitoba, Canada; *G.breviceps* collected in Oklahoma and Texas; and *Geomyspersonatus* True, collected in Texas.

Hall (1916) described the following nematodes from *Thomomysfossor* J. A. Allen [syn. *T.talpoides* (Richardson, 1828)]: *Trichurisfossor* Hall, 1916, from specimens collected near both Crested Butte and Livermore, Colorado and *Vexillatavexillata* (Hall, 1916) from gophers collected from mountain meadows near Livermore, Colorado. These nematodes were described from the same material that Hall (1912) had previously studied. Additionally, the nematode *Protospiruraascaroidea* Hall, 1916 was described from specimens recovered from the stomachs of *Geomysbursarius* collected near Norman, Oklahoma by Herman Douthitt and sent to MC Hall for study.

Skidmore (1929) described a species of Coccidia named *Eimeriageomydis* Skidmore, 1929 from the intestinal tract of *Geomysbursarius* Shaw, collected near Lincoln, Nebraska while Dikmans (1932) reported *Capillaria* (syn. *Calodium*) *hepaticum* (Bancroft, 1893) as a parasite of *Thomomysfossor* (syn. *T.talpoides*) collected in the Medicine Bow Mountains of Wyoming. In that same year, English (1932) examined 161 specimens of *Geomysbursarius* collected in Brazos County, Texas and found 23 infected with the stomach nematode *Protospiruraascaroidea* Hall, 1916, and eight infected with an unknown species of *Hymenolepis*.

Hubbell and Goff (1939) reported *Mastophorusmurisascaroides* (Gmelin, 1790) to occur commonly in the stomach of *Geomys* sp. (most likely *G.pinetis*) collected near Leesburg, Lake County, Florida.

McIntosh (1941) described *Catenotaenialinsdalei* McIntosh, 1941 from *Thomomysbottaebottae* (Eydoux & Gervais, 1836) collected near Monterey, California on the Hastings Natural History Reservation.

Caballero and Cerecero (1943) described *Vexillataconvoluta* from the small intestine of the Merriam’s pocket gopher, *Cratogeomysmerriami* (Thomas, 1893), collected from the state of Michoacan, Mexico.

Chandler (1945) redescribed *Trichurisfossor* Hall, 1916 from *Thomomysbottaebottae* from specimens collected on the Hastings Natural History Reservation near Monterey, California. This was the first good description of the eggs of *T.fossor*, and the first report of *T.fossor* from *T.bottae*. In the same year during an ecological study of the small mammals collected from Northrup Canyon in eastern Washington State, Rankin (1945) recorded *Hymenolepisdiminuta* (Rudolphi, 1819) from *Thomomystalpoides*, see discussion below. The next year, Wenrich (1946) recorded a species of *Monocercomonoides* Travis, 1932 as a cecal commensal (flagellate) of Botta’s pocket gopher, *Thomomysbottae*.

Tryon (1947) reported both cestodes and nematodes in *Thomomystalpoides* from Montana, with most of his field work occurring in the Bridger Mountains. Less than one percent of the gophers necropsied contained an unidentified species of cestode. Nematodes identified as belonging to the family Trichuridae were found in 100% of the pocket gophers examined for endoparasites. In areas of low pocket gopher density, the prevalence of infection was low (approximately 10%); however, in areas of high gopher density, the prevalence of infection approached 80%. Tryon (1947) speculated that the young gophers became infected before leaving the parental burrows, and by August, the prevalence of infection for the young pocket gophers was ca. 50%. Nematodes, probably of the genus *Protospirura* were found in the stomachs of some gophers, with as many as 42 in an individual pocket gopher’s stomach. Concerning the presence of warbles in the pocket gophers examined during the study, Tryon stated “only 15 out of over a thousand animals examined showed warbles. Of these, ten were juveniles indicating that they may be above ground more than the adults, probably during migration from the parental burrows.”

Rausch and Schiller (1949), during a study of cestodes of the genus *Andrya* Railliet, 1893, mentioned *Andryamacrocephala* Douthitt, 1915 as occurring in *Thomomystalpoidestenellus* Goldman from the Jackson Hole Wildlife Park in Wyoming.

Hansen (1950), during a study of the tapeworms of rodents, recorded *Andryamacrocephala* Douthitt, 1915 as occurring in 5 of 5 *Geomysbursarius* examined with up to 12 cestodes per host. Hansen (1950) also recorded *Monoecocestusanoplocephaloides* (Douthitt, 1915) from *Thomomysbottae* collected in the region of Sacramento, California. Interestingly, this cestode has not since been reported from any members of the genus *Thomomys*.

Smith (1951), in a study of the cestodes of *Thomomystalpoides* collected from Carbon County, Wyoming, reported the following cestodes: *Pseudocittotaeniapraecoquis* (Stiles, 1895) from the small intestine; *P.megasacca* (Smith, 1951) also from the small intestine (see below for clarification of the taxonomy of these two species). Smith (1951) also included a list of the cestodes reported from pocket gophers up to that time and attempted to clarify the taxonomic relationships between *Schizotaenia* Janicki, 1904 and *Monoecocestus* Beddard, 1914.

Ingles (1952) reported *Trichuris* sp. (probably *T.fossor*) as a common parasite of the cecum of *Thomomysmonticola* J. A. Allen, 1893. All specimens that Ingles examined came from an elevation of ca. 7,000 feet in the Sierra Nevada of California. In the same year, Everett Schiller (1952), in a study of the morphological variation in *Hymenolepis* (syn. *Arostrilepis*) *horrida* (von Linstow, 1901) reported *Thomomysbottae* from near O’Neals California as a host.

Burnham (1953), during a study of the parasites of *Geomysbursarius*, collected from four counties in Oklahoma reported the following species of parasites: *Protospiruramurisascaroides* (Hall, 1916) (syn. *Mastophorusmuris*) from the stomachs of 18 hosts; *Litomosafilaria* Beneden, 1897 from the pleural cavities of 19 gophers (this is probably a misidentification, see discussion below regarding the filarioid nematodes of pocket gophers); *Ostertagia* sp. from the stomachs of five gophers; *Hymenolepisdiminuta* (Rudolphi, 1819) from the small intestines of ten hosts (see discussion below for clarification of the problem concerning *H.diminuta* in geomyids); *Monoecocestusanoplocephaloides* (Douthitt, 1915) from 25 hosts, with a range of infection of 1–100 worms per host; and *Cittotaeniaperplexa* Stiles, 1897 from two gophers.

Soon after, Smith (1954) described *Hymenandryathomomyis* from the small intestine of *Thomomystalpoides* collected in Colorado and in this same publication, he recommended that *Catenotaenialinsdalei* McIntosh, 1941 be considered a synonym of *C.dendritica* (Goeze, 1782) Janicki 1904.

Voge (1955) in a catalogue of the cestode parasites of California mammals, listed *Catenotaeniadendritica* (Goeze, 1782), *Hymenolepiscitelli* (McLeod, 1933), and an unidentified species of *Hymenolepis* from *T.bottae*.

The next year, Voge (1956), in a list of the nematode parasites of California mammals, reported *Trichurisfossor* Hall, 1916 as a parasite of *T.bottae* and in the same year, Lubinsky (1956) reported *Calodium* (syn. *Capillaria*) *hepaticum* from *T.talpoides* in Alberta, Canada. Soon after, continuing his work on small mammals, Lubinsky (1957) in a list of the helminth parasites of rodents from Alberta included the following as parasites of *Thomomystalpoides*: *Versteria* (syn. *Taenia*) *mustelae* (larvae) from the mesenteries, lungs, liver, and kidneys of gophers collected in northern and middle Alberta: *Anoplocephaloidesvariabilis* (Douthitt, 1915) recovered from the colon (which is a doubtful location for a cestode) from six localities in middle and southern Alberta; *Calodium* (syn. *Capillaria*) *hepaticum* from the livers of gophers collected from central and southern Alberta; *Trichurisfossor* from the cecum of gophers collected from central Alberta; *Protospiruraascaroidea* Hall, 1916 from the stomachs of gophers from middle Alberta; *Litomosoidescarinii* (Travassos, 1919) from the coelom of pocket gophers from middle and southern Alberta. In the same year, Levine, et al. (1957) described *Eimeriathomomysis* from specimens of *T.bottae* collected in the Grand Canyon of Arizona.

Howard and Childs (1959) during a study of the ecology of *Thomomysmonticola* reported *Hymenolepishorrida* (von Linstow, 1901) to occur commonly in adult pocket gophers. They stated, “Most of the adults had several tapeworms (*Hymenolepishorrida*), and one animal had 108 immature tapeworms with short strobila. None of the five juvenile gophers examined had tapeworms.” Based on recent work by Dursahinhan et al. (2022), it appears now that the species identified as *H.horrida* may be referred to the genus *Arostrilepis*.

Frandsen and Grundmann (1960) discussed the geographic distribution of *Trichurisfossor* Hall, 1916 and *Ransomusrodentorum* Hall, 1916 from *Thomomystalpoides* and *T.umbrinus* in the Lake Bonneville basin of Utah. They speculated that the distribution of these two species of nematodes in *Thomomys* sp. in this area supports the contention that competition occurred between the two species of pocket gophers resulting in the present-day distribution patterns of the pocket gophers and their respective helminths.

Rausch (1961) reported *Calodium* (syn. *Capillaria*) *hepaticum* from *Thomomystalpoidestenellus* Goldman from near Moran, Wyoming, collected in June of 1948 and Frandsen and Grundmann (1961) reported the following helminth parasite species from several subspecies of both Northern pocket gopher, *Thomomystalpoides*, and the Southern pocket gopher *Thomomysumbrinus* (Richardson, 1829). These species include *Ascarislaevis* Leidy, 1856, *Hymenolepiscitelli*, *Ransomusrodentorum*, and *Trichurisfossor*. However, *Anoplocephaloidesinfrequens* (Douthitt, 1915), *A.variabilis* (Douthitt, 1915), *Pseudocittotaeniapraecoquis* (Stiles, 1985), *Arostrilepishorrida*, and *Nippostrongylusmuris* (Yokogawa, 1920) have been reported from *T.talpoides*. In addition, *Paruterinacandelabraria* (Goeze, 1781) and *Moniliformisclarki* are only reported from *T.umbrinus*.

Stock (1962) reported three males and one female of the nematode *Ransomusrodentorum* from the cecae of two specimens of *Thomomystalpoidesfossor*, collected at the junction of Dry Gulch and the Gunnison River, Colorado, at ca. 7,400 feet altitude.

Rissky (1962) reported *Monocercomonoides* from the cecum of the Plains pocket gopher, *Geomysbursarius*, collected from Clay County, South Dakota.

Ubelaker and Downhower (1965) in a study of the endo and ectoparasites of *Geomysbursarius* in Kansas, reported *Calodium* (syn. *Capillaria*) *hepaticum* from the cecum of a single pocket gopher and *Andryamacrocephala* Douthitt, 1915 and *Anoplocephaloidesinfrequens* (Douthitt, 1915) were found to occur in seven and six of the pocket gophers examined, respectively.

Lainson (1968), during a parasitological study in El Cayo District British Honduras, a new species of coccidian parasite (*Eimeriaorthogeomyos*) was described from the Giant pocket gopher, *Orthogeomysgrandis* (Thomas, 1893) collected from Baking Pot, El Cayo District, Central America (Lainson, 1968).

Tryon and Cunningham (1968) in a study of *Thomomystalpoides* along an altitudinal transect in the Beartooth Mountains of Wyoming reported *Calodium* (syn. *Capillaria*) *hepaticum* from the livers of 5%, 37%, and 8% of the gophers from the Alpine, the Canadian, and the transition life zones, respectively.

Douglas (1969) studied the ecology of the pocket gophers of Mesa Verde, Colorado. He reported *Trichurisfossor* Hall, 1916 and Cuterebracf.cyanella (bot fly larvae) from *Thomomysbottaeaureus*Douglas (1969) stated that, “Of the gophers infected with bot fly larvae, the highest prevalence of infection occurred during September, with no gophers carrying larvae during the spring.” Douglas (1969) also stated “Specimens of Cestoda currently are being studied and will be reported elsewhere.” To our knowledge, no report has ever been published.

Todd and Tryon (1970) described *Eimeriafitzgeraldi* Todd & Tryon, 1970 from *Thomomystalpoides* collected from the Beartooth Mountains, Park County Wyoming. Oocysts were recovered from the feces of two of ten juvenile males and one of 31 adult females (pocket gophers).

Todd et al. (1971) in a study of the endoparasites of the Northern pocket gopher (*Thomomystalpoides*) from Park County, Wyoming, reported the following species of parasites from a total of 46 specimens of *T.talpoides* examined: *Eimeriathomomysis* Levine, Ivens & Kruidenier, 1957 was found to occur in the fecal pellets of 24 of the individual gophers; *E.fitzgeraldi* Todd & Tryon, 1970 was found in the feces of two gophers; cestode cysticerci of the family Taeniidae were found in the mesenteries near the stomach and cecum of one gopher; fragments of the cestode *Catenotaenialinsdalei* McIntosh, 1941 were found in the body cavities of two animals (this is a dubious body location record); *Anoplocephaloidesvariabilis* (Douthitt, 1915) was present in the small intestines of 18 gophers; *A.infrequens* (Douthitt, 1915) was recovered from the small intestine of seven gophers; *Anoplocephaloides* sp. was recovered from the small intestines of 22 gophers *R.rodentorum* was found in the cecum of 34 gophers, and in the large intestine of one; *Vexillatavexillata* was recovered from the small intestines of two gophers; *Protospiruraascaroidea* was found in the stomachs of two animals; *Trichurisfossor* was found in the ceca of 30 gophers; and *Calodium* (syn. *Capillaria*) *hepaticum* was recovered from the livers of 18 of the gophers examined.

Todd and Lepp (1972) redescribed *Trichurisfossor* from specimens recovered from *T.talpoides* from Park County, Wyoming.

Grundmann et al. (1976), in a paper discussing the mechanisms of parasitic helminth population regulation in rodents, listed the following parasites as occurring in *Thomomystalpoides* in Utah: *Trichurisfossor*, *Vexillatavexillata*, *Ascarislaevis* Leidy, 1856. *Hymenolepishorrida*, and *T.fossor* were reported from *T.bottae* in the same paper.

Rausch (1976) in a study of the rodent cestode genera *Paranoplocephala* Luhe, 1910 and *Anoplocephaloides* Baer, 1923 examined the type material of *Anoplocephaloidesinfrequens* (Douthitt, 1915) from *Geomysbursarius* collected by Douthitt in Brainerd, Minnesota, and specimens of *A.variabilis* (Douthitt, 1915) collected by Douthitt in central Illinois from *Geomysbursarius*. Also studied by Rausch (1976) were seven specimens of *A.variabilis* from *Thomomystalpoides* collected at Emerson, Manitoba, 10 km north of Prince Albert, Saskatchewan, Canada and from 5 km south of Saskatoon, Saskatchewan, Canada. Rausch (1976) stated “I also obtained it (*A.variabilis* from *T.talpoides*) in two of 11 of these rodents at Moran, Wyoming, in 1949.”

Beveridge (1978) in a revision of the genus *Pseudocittotaenia* Tenora, 1976, listed the synonyms of *P.praecoquis* (Stiles, 1895) and described *P.glandularis* Beveridge, 1978 from some specimens taken from *Thomomystalpoides* in Utah by Frandsen and Grundmann (1961), and from some specimens from *T.talpoides* in Wyoming. Frandsen and Grundmann (1961) evidently misidentified *P.glandularis* Beveridge, 1978 and had determined that the specimens that they found in *T.talpoides* were *Pseudocittotaeniapraecoquis* (Stiles, 1895). The specimens from the Wyoming pocket gophers were from material that Smith (1951) had mistakenly identified and redescribed as *P.praecoquis*. Beveridge (1978) also listed as synonyms: *P.megasacca* (Smith, 1951) with *P.praecoquis* (Stiles, 1895). Also reported by Beveridge (1978) and not reported elsewhere in the literature was *Pseudocittotaeniapraecoquis* from *T.talpoidestenellus* Goldman, collected by Robert L. Rausch near Moran, Wyoming in June of 1948.

Jasmer (1980) in a thesis written at Humboldt State University listed the following parasites from *Thomomysbottae* (Eydoux & Gervais): *Ransomusrodentorum*, *Trichurisfossor*, *Hymenolepiscitelli*, and an unidentified species of *Heligmosomoides* Hall, 1916. He also discussed the biological characteristics and taxonomy of *R.rodentorum* (some of his specimens are now in the Manter Laboratory Parasite Collection).

Gardner and Jasmer (1983) described *Heligmosomoidesthomomyos* Gardner & Jasmer, 1983 from *Thomomysbottae* (Eydoux & Gervais) and *T.bulbivorus* (Richardson) from Humboldt County, California and Benton County, Oregon, respectively. They included some measurements and remeasurements of two other species of *Heligmosomoides*: *H.longispiculatus* (Dickmans, 1940) and *H.montanus* Durette-Desset, 1968.

Gardner (1985) described *Hymenolepistualatinensis* from the duodenum of the Camas pocket gopher, *Thomomysbulbivorus* (Richardson, 1829) collected near the Tualatin River in the Willamette Valley of Oregon. In the report, several helminth species were documented during the study including *Arostrilepishorrida* also from the small intestine, *Trichurisfossor* from the cecum, *Ransomusrodentorum* from the cecum, and *Heligmosomoidesthomomyos* from the duodenum.

Gardner and Schmidt (1986) described *Litomosoidesthomomydis* from the abdominal cavity of the Northern pocket gopher, *Thomomystalpoides*, and Botta’s pocket gopher, *Thomomysbottae*, from Huerfano County, Colorado. Also, *L.westi* was described from the abdominal and pleural cavities of the Plains Pocket Gopher, *Geomysbursarius*, collected from Weld County, Colorado.

Shortly after this work, Gardner and Schmidt (1988) described two new species in the genus *Hymenolepis* Weinland, 1858, including *H.weldensis* and *H.geomydis* from the small intestines (duodenum) of the Plains pocket gopher, *Geomysbursarius*, collected from Weld County, Colorado.

Pitts et al. (1990) reported *Litomosoideswesti* Gardner & Schmidt, 1986 from *Geomyspersonatus* True, 1889 collected from Duval and Zapata counties in Texas.

Upton et al. (1992) reported *Eimeriageomydis* Skidmore, 1929 from Baird’s pocket gopher, *Geomysmericanu*, and Llano pocket gopher, *Geomystexensis* Merriam, 1895 collected from Texas.

Dronen et al. (1994) described *Monoecocestuscentroovarium* found in Attwater’s pocket gopher, *Geomysattwateri* Merriam, 1895 collected from Atascosa County, Texas. In the same year, Wilber et al. (1994) described *Eimeriajemezi* found in the Northern pocket gopher, *Thomomystalpoides* collected from El Cajete crater, Jemez Springs, Sandoval County, New Mexico.

Lamothe-Argumedo et al. (1997) reported *Paraspidoderamerican* Travassos, 1914 from the intestine of Merriam’s pocket gopher, *Cratogeomysmerriami* (Thomas, 1893) first collected from Morelos, Cuernavaca, Mexico in 1984.

Pitts et al. (2000) reported the additional occurrence of the filarioid nematode, *Litomosoideswesti* from the pleural cavities of Baird’s pocket gopher, *Geomysmericanu* collected at the entrance of Isle, Du Boris unit, Lake Ray Roberts State Park, Denton County, Texas while *L.westi* was also documented from the pleural cavities of the Plains pocket gopher, *Geomysbursarius* captured near Aubrey, Grubbs Road, same county.

Bartel and Gardner (2000) reported the helminth parasites from the Plains pocket gopher, *Geomysbursarius*, from seven localities in the northern boundary range, Minnesota. The report includes the following: *Physalopteralimbata* Leidy, 1856 from the stomach, *Ransomusrodentorum* from the cecum and large intestine and *Calodium* (syn. *Capillaria*) *mericanum* (Read, 1949), *Anoplocephaloidesinfrequens*, *A.variabilis* (Douthitt, 1915), *Andryamacrocephala*, *Hymenolepisweldensis* Gardner & Schmidt, 1988 and *Moniliformisclarki* from the small intestines.

Falcón-Ordaz et al. (2006) described *Vexillatageomyos* from Attwater’s pocket gopher, *Geomysattwateri* from the Welder Wildlife Refuge of San Patricio County, Texas.

Using molecular methods, Haukisalmi et al. (2010) documented *Hymenolepisweldensis* from *Geomysbursarius* collected from Illinois and Indiana.

Makarikov et al. (2012) described *Arostrilepisschilleri* obtained from the Camas pocket gopher, *Thomomysbulbivorus*, captured southeast of Corvallis, Oregon and originally reported as *H.horrida* by Gardner (1985).

LeBrasseur (2017) in an unpublished master’s thesis reported a study focused on the endoparasites of four species of pocket gophers in the genus *Geomys* collected from eight counties in Texas. These host species included the Plains pocket gopher, *Geomysbursarius*, Attwater’s pocket gopher, *G.attwateri* Merriam, 1895, Texas pocket gopher, *G.personatus* True, 1889, and the Central Texas pocket gopher *G.texensis* Merriam, 1895. In addition, she found an unidentified *Hymenolepis* Weinland, 1858 obtained from *G.attwateri*, *G.bursarius*, and *G.texensis* and another tapeworm, *Monoecocestus* was obtained from *G.bursarius*¸ and *G.texensis*. Finally, a nematode species, *Protospiruraascaroidea*, was found from all four species of *Geomys* mentioned above; the specimens were verified by HWML personnel (LeBrasseur 2017).

Gardner et al. (2020) described two new species of unarmed hymenolepidid tapeworms, including *Hobergiairazuensis* from the small intestine of *Heterogeomysheterodus* (Peters, 1865), collected from Potrero Cerrado, Cartago, Costa Rica, and *Hymenolepiscratogeomyos* from the small intestine of the Volcán De Toluca pocket gopher, *Cratogeomysplaniceps* (Merriam, 1895) collected from Parque Nacional Nevado de Toluca, México. Also, *H.weldensis* Gardner & Schmidt, 1988 has been documented from many individuals of *Geomyslutescens* Merriam, 1890 collected in the Sandhills, on the north side of the North Platte River near Cedar Point Biological Station in western Nebraska.

The present study reports an unidentified *Monoecocestus* sp. Beddard, 1914 (probably *M.anoplocephaloides*) from the small intestine of the Yellow-faced pocket gopher, *Cratogeomyscastanops* (Baird, 1852), collected by a local landowner from Black Mesa, Oklahoma in 2016 (NP2779). *Anoplocephaloidesvariabilis* (Douthitt, 1915) was found from the small intestine of Hall’s pocket gopher, *Geomysjugossicularis* Hooper, 1940 collected from Grama grass habitat, Keith County, Nebraska in 2016 (NP2661). Also, from 2009–2016, necropsies of *Geomyslutescens* Merriam, 1890 yielded many individuals of *Litomosoideswesti* Gardner & Schmidt, 1986 from their abdominal cavities with individuals of *Ransomusrodentorum* from the cecum, and from two pocket gophers *Physalopteralimbata* Leidy, 1856 was found (NP2297, NP2298). Also, during general collecting in the area of Nebraska, we found two nematode species (*R.rodentorum*, and *T.fossor* – refer to NP1524) from the cecum of the Wyoming pocket gopher, *Thomomysclusius* Coues, 1875, collected from 5 miles east of Woods Landing, Albany County, Wyoming in 2013. All specimens mentioned in this work are deposited in the HW Manter Laboratory of Parasitology Museum collection where NP refers to the field collection number.

#### ﻿Endoparasites of Neotropical subterranean rodents

**Figure 4.**
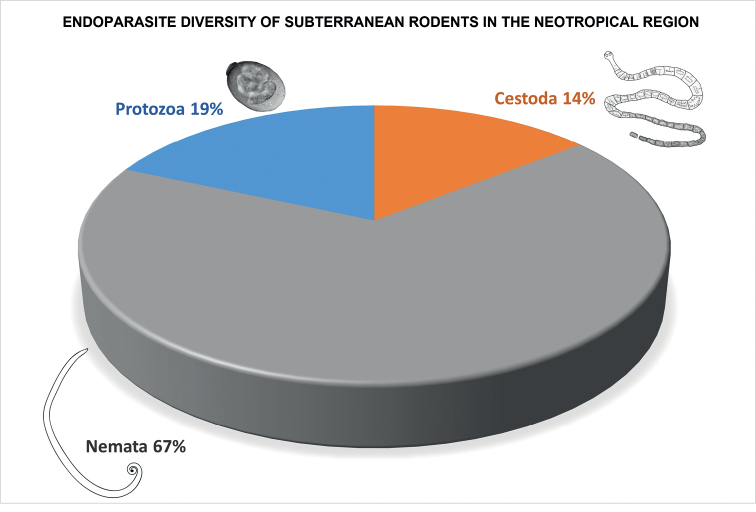
Percentage taxon composition of endoparasite diversity pie diagram shown by higher classification of bothprotozoa and helminths occurring in subterranean rodents (Family Ctenomyidae) in the southern Neotropical region. All records of parasites presented were derived from a review of the literature published from 1931 through 2021. Approximately 67% of the total endoparasite fauna of these rodents consists of Nemata, followed by Protozoa (19%), and Cestoda (14%).

See graphical summary in Fig. 4 and endoparasite list Table 5.

**Table 5.** Endoparasite species diversity from Neotropical subterranean rodents (Ctenomyidae and Octodontidae). Authorities are given for parasite and host species.

Host species	Parasite species	References
*Ctenomysandersoni* Gardner, et al., 2014	*Paraspidodera* Travassos, 1914	Gardner et al. 2021
*Ctenomysaustralis* Rusconi, 1934	*Pudicactenomydis* Rossin et al., 2006	Rossin et al. 2010a
	*Taeniatalicei* Dollfus, 1960	Rossin et al. 2010b
	*Trichurispampeana* Suriano & Navone, 1994	Rossin et al. 2010a
*Ctenomysazarae* Thomas, 1903	*Trichurispampeana* Suriano & Navone, 1994	Suriano and Navone 1994; Rossin and Malizia 2005a
*Ctenomysboliviensis* Waterhouse, 1848	*Ancylostomactenomyos* Drabik & Gardner, 2019	Drabik and Gardner 2019
	*Paraspidodera* Travassos, 1914	Gardner et al. 2021
	*Eimeriaopimi* Lambert et al., 1988	Gardner and Duszynski 1990
*Ctenomysconoveri* Osgood, 1946	*Eimeriaopimi* Lambert et al., 1988	Gardner and Duszynski 1990
	*Paraspidodera* Travassos, 1914	Gardner et al. 2021
*Ctenomyserikacuellarae* Gardner et al., 2014	*Paraspidodera* Travassos, 1914	Gardner et al. 2021
*Ctenomysandersoni* Gardner, et al., 2014	*Paraspidodera* Travassos, 1914	Gardner et al. 2021
*Ctenomysaustralis* Rusconi, 1934	*Pudicactenomydis* Rossin et al., 2006	Rossin et al. 2010a
	*Taeniatalicei* Dollfus, 1960	Rossin et al. 2010b
	*Trichurispampeana* Suriano & Navone, 1994	Rossin et al. 2010a
*Ctenomysazarae* Thomas, 1903	*Trichurispampeana* Suriano & Navone, 1994	Suriano and Navone 1994; Rossin and Malizia 2005a
*Ctenomysboliviensis* Waterhouse, 1848	*Ancylostomactenomyos* Drabik & Gardner, 2019	Drabik and Gardner 2019
	*Paraspidodera* Travassos, 1914	Gardner et al. 2021
	*Eimeriaopimi* Lambert et al., 1988	Gardner and Duszynski 1990
*Ctenomysconoveri* Osgood, 1946	*Eimeriaopimi* Lambert et al., 1988	Gardner and Duszynski 1990
	*Paraspidodera* Travassos, 1914	Gardner et al. 2021
*Ctenomyserikacuellarae* Gardner et al., 2014	*Paraspidodera* Travassos, 1914	Gardner et al. 2021
	*Raillietina* Fuhrman, 1920	Gardner et al. 2021
*Ctenomysfrater* Thomas, 1902	*Eimeriaopimi* Lambert et al., 1988	Gardner and Duszynski 1990
	*Paraspidodera* Travassos, 1914	Gardner et al. 2021
*Ctenomysfulvus* Philippi, 1860	*Trichurisfulvi* Babero & Murua, 1987	Babero and Murua 1987
	*Trichurisrobusti* Babero & Murua, 1990	Babero and Murua 1990
*Ctenomyslewisi* Thomas, 1926	*Eimeriaopimi* Lambert et al., 1988	Gardner and Duszynski 1990
	*Paraspidodera* Travassos, 1914	Gardner et al. 2021
*Ctenomysleucodon* Waterhouse, 1848	*Pudicapujoli* Durette-Casset & Tcheprakoff, 1990	Gardner et al. 2021
*Ctenomysmagellanicus* Bennett, 1836	*Paraspidoderaamericana* Khalil & Vogelsang, 1931	Khalil and Vogelsang 1931
*Ctenomysmaulinus* Philippi, 1872	*Monoecocestustorresi* Olsen, 1976	Olsen 1976
	*Paraspidoderauncinata* Rudolphi, 1819	Torres et al. 1976
	*Trichuris* Roederer, 1761	Torres et al. 1976
*Ctenomysnattereri* Wagner, 1848	*Paraspidodera* Travassos, 1914	Gardner et al. 2021
	*Trichuris* Roederer, 1761	Gardner et al. 2021
*Ctenomysopimus* Wagner, 1848	*Eimeriagranifera* Lambert et al., 1988	Lambert et al. 1988; Gardner and Duszynski 1990
	*Eimeriamontuosi* Lambert et al., 1988	Lambert et al. 1988; Gardner and Duszynski 1990
	*Eimeriaopimi* Lambert et al., 1988	Lambert et al. 1988; Gardner and Duszynski 1990
	*Eimeriaoruroensis* Lambert et al., 1988	Lambert et al. 1988; Gardner and Duszynski 1990
	*Litomosoidesandersoni* Brant & Gardner, 1997	Brant and Gardner 1997
	*Litomosoidesctenomyos* Brant & Gardner, 1997	Brant and Gardner 1997
	*Mathevotaenia* Akhumyan, 1946	Gardner et al. 2021, 2023
*Ctenomyspearsoni* Lessa & Langguth, 1983	*Strongyloidesmyopotami* Artigas & Pacheco, 1933	Rossin et al. 2009
*Ctenomyssteinbachi* Thomas, 1907	*Ancylostomactenomyos* Drabik & Gardner, 2019	Drabik and Gardner 2019
	*Eimeriaopimi* Lambert et al., 1988	Gardner and Duszynski 1990
	*Paraspidodera* Travassos, 1914	Gardner et al. 2021
*Ctenomystalarum* Thomas, 1898	*Graphidiodessubterraneus* Rossin et al., 2005	Rossin et al. 2005b; Rossin et al. 2010b
	*Heligmostrongylus* Travassos, 1917	Rossin and Malizia 2002
	*Paraspidoderauncinata* Rudolphi, 1819	Rossin et al. 2004b; Rossin et al. 2010b
	*Pudicactenomydis* Rossin et al., 2006	Rossin et al. 2006a; Rossin et al. 2010b
	*Strongyloidesmyopotami* Artigas & Pacheco, 1933	Rossin et al. 2010b; Rossin et al. 2009
*Ctenomystalarum* Thomas, 1898	*Hydatigera* (syn. *Taenia*) *taeniaeformis* Batsch, 1786	Rossin et al. 2004a
	*Taeniatalicei* Dollfus, 1960	Rossin et al. 2010a; Rossin et al. 2010b
	*Trichostrongylusduretteae* Rossin et al., 2006	Rossin et al. 2006b; Rossin et al. 2010a
	*Trichuris* Roederer, 1761	Rossin and Malizia 2002; Rossin and Malizia 2005a
	*Trichurisbursacaudata* Suriano & Navone, 1994	Suriano and Navone 1994
	*Trichurispampeana* Suriano & Navone, 1994	Rossin et al. 2010a; Rossin and Malizia 2005a
*Ctenomystorquatus* Lichtenstein, 1830	*Taeniatalicei* Dollfus, 1960	Dollfus 1960
*Spalacopuscyanus* (Molina, 1782)	*Graphidioides yañezi* Babero & Cattan, 1980	Babero and Cattan 1980

Khalil and Vogelsang (1931) described the first helminth parasite from a subterranean host from Neotropical region, *Paraspidoderaamericana* Khalil & Vogelsang, 1931 from the cecum of a single individual of what they called *Ctenomysmagellanicus* Bennett, 1836 collected from Carrasco near Montevideo, Uruguay in 1927. The identification of this mammal specimen was probably erroneous as *C.magellanicus* occurs only near the Strait of Magellan in southern Argentina). Unfortunately, no hosts or parasite specimens were deposited in any collection that we can find up to the current time.

Dollfus (1960) described *Taeniatalicei* Dollfus, 1960 from the abdominal cavity of the Collared tuco-tuco, *Ctenomystorquatus* Lichtenstein, 1830, collected from Uruguay and in 1986, multistrobilate larvae of *T.talicei* were collected from several *Ctenomysopimus* at 7 km S: 4 km E. Cruce Ventilla, Oruro, Bolivia by a party from the American Museum of Natural History and the Museum of Southwestern Biology (Anderson 1997).

Olsen (1976) described *Monoecocestustorresi* obtained from the small intestine of Maule’s tuco-tuco, *Ctenomysmaulinus* Philippi, 1872 collected near Lonquimay, Chile. Meanwhile, Torres et al. (1976) reported *Paraspidoderauncinata* Rudolphi, 1819, and unidentified *Trichuris* are reported from Maule’s tuco-tuco, *Ctenomysmaulinus*, collected from Chile.

Babero and Cattan (1980) described *Graphidiodes yañezi* from the small intestine of a coruro, *Spalacopuscyanus* (Rodentia: Octodontidae), collected from near Concón, Chile.

Babero and Murua (1987) described a new species of whipworm, *Trichurisfulvi* obtained from the cecum of the Tawny tuco-tuco, *Ctenomysfulvus* Philippi, 1860, collected from San Pedro Atacama, Tarapaca province, Chile.

Lambert et al. (1988) described four new coccidian parasites in the genus *Eimeria* Schneider, 1875 recovered from the feces of the Highland tuco-tuco, *Ctenomysopimus* Wagner, 1848, trapped from several localities of the Department of Oruro, Bolivia, South America. Those species are *E.granifera* from Rancho Huancaroma, near the Rio Desaguadero, *E.montuosi*, from the north of Pomata Ayte, Rio Barros, *E.opimi*, and *E.oruroensis*, from the northeast and east of Rancho Huancaroma.

Babero and Murua (1990) described *Trichurisrobusti* from the cecum and large intestine of the Tawny tuco-tuco, *Ctenomysfulvus*, collected from La Hauyca, Tarapaca province, Chile.

Gardner and Duszynski (1990), during a study on morphometric comparison of a coccidian species, *Eimeriaopimi*Lambert et al., 1988, in different regions of Bolivia, the following host species were detected positive for this protozoan parasite. Those hosts include Lewis’s tuco-tuco, *Ctenomyslewisi* Thomas, 1926, collected from the areas of the high-altitude region in Tarija; the Reddish tuco-tuco, *Ctenomysfrater* Thomas, 1902, collected from medium latitude region of Tarija; the Conover’s tuco-tuco, *Ctenomysconoveri* Osgood, 1946, collected from Chaco thorn forest area in Chuquisaca; the Bolivian tuco-tuco, *Ctenomysboliviensis* Waterhouse, 1848, and the Steinbach’s tuco-tuco, *Ctenomyssteinbachi* Thomas, 1907 collected from the Tropical palm/savanna region of Santa Cruz, Bolivia. In addition, the following coccidian parasites were reported from the Highland tuco-tuco, *Ctenomysopimus*. These species include *Eimeriaopimi*, *E.granifera*Lambert et al., 1988, *E.oruroensis*Lambert et al., 1988, and *E.montuosi*Lambert et al., 1988 collected from the Oruro region *E.opimi* and *E.granifera* collected from the Potosi region.

Suriano and Navone (1994) described *Trichurisbursacaudata* obtained from the cecum of the Talas tuco-tuco, *Ctenomystalarum* Thomas, 1898 collected from Punta Indio, Buenos Aires, and *T.pampeana* found in the cecum of the Azara’s tuco-tuco, *Ctenomysazarae* Thomas, 1903, collected from Santa Rosa, La Pampa, Argentina (Suriano and Navone 1994). However, *T.pampeana* has been redescribed from its original voucher specimens (Rossin and Malizia 2005).

Brant and Gardner (1997) described *Litomosoidesandersoni* and *L.ctenomyos* from the abdominal and thoracic regions of the Highland tuco-tuco, *Ctenomysopimus*, collected from near Rancho Huancaroma, Departamento de Oruro, Bolivia.

Rossin and Malizia (2002), during a study of the relationship between helminth parasites and demographic attributes of a population, two unidentified helminth parasites were reported. Those include *Heligmostrongylus* Travassos, 1917 found in the small intestine, and *Trichuris* recovered from the cecum of the Talas tuco-tuco, *Ctenomystalarum*, collected from Necochea, Buenos Aires province, Argentina.

Rossin et al. (2004a) reported larvae of *Hydatigera* (syn. *Taenia*) *taeniaeformis* from the peritoneal cavity and liver of the Talas tuco-tuco, *Ctenomystalarum*, trapped in the urban areas of Mar de Cobo, Buenos Aires province, Argentina. These authors experimentally infected dogs with this species of tapeworm from the tucos and recovered adult cestodes.

Rossin et al. (2004b) redescribed *Paraspidoderauncinata* (Rudolphi, 1819) from a large number of specimens obtained from the cecum and large intestine of the Talas tuco-tuco, *Ctenomystalarum*, collected from Mar de Cobo, Buenos Aires province, Argentina.

Rossin and Malizia (2005a) redescribed *Trichurispampeana* Suriano & Navone, 1994 found in the cecum of the Azara’s tuco-tuco, *Ctenomysazarae*, collected from Santa Rosa, La Pampa province, and reported new voucher material, the Talas tuco-tuco, *C.talarum* Thomas, 1898, collected at the Necochea, coastal dunes of Buenos Aires province. Also, an unidentified *Trichuris* found in *C.talarum* collected from Buenos Aires province, Argentina was reported. Simultaneously, Rossin et al. (2005b) described *Graphidiodessubterraneus* from the stomach of the Talas tuco-tuco, *Ctenomystalarum*, collected from Mar de Cobo, Partido de Mar Chiquita, Mar del Plata, Argentina.

Continuing work on tucos, Rossin et al. (2006a) described *Pudicactenomydis* from the small intestine of the Talas tuco-tuco, *Ctenomystalarum*, collected from Mar de Cobo, Partido de Mar Chiquita, Argentina. In the same year, Rossin et al. (2006b) described *Trichostrongylusduretteae* obtained from the small intestine of the Talas tuco-tuco, *Ctenomystalarum*, collected from Mar de Cobo, Buenos Aires province, Argentina.

Rossin et al. (2009) reported *Strongyloidesmyopotami* Artigas & Pacheco, 1933 found in the small intestines of the Talas tuco-tuco, *Ctenomystalarum*, collected from Mar de Cobo, Buenos Aires province, Argentina, and from Pearson’s tuco-tuco, *Ctenomyspearsoni* Lessa & Langguth, 1983, collected from Penino, Departamento de San José, Uruguay.

During an ecological study of helminth parasite infection parameters in two species of South American subterranean rodents of the genus *Ctenomys*, Rossin et al. (2010a) documented seven species of Endoparasites from two collection localities, species of hosts studied included the Southern tuco-tuco, *C.australis* Rusconi, 1934, from Necochea, Buenos Aires Province, and Talas tuco-tuco, *C.talarum* Thomas, 1898, from Mar de Cobo, Buenos Aires province, Argentina. Both species of tuco-tuco’s harbored *Trichurispampeana* in the cecum, *Pudicactenomydis* Rossin et al., 2006 in the small intestine, and larvae of *Taeniatalicei* in the abdominal cavity. Moreover, *C.talarum* had four additional species of helminths, including *Graphidiodessubterraneus* Rossin et al., 2005 in the stomach, *Paraspidoderauncinata* in the large intestine, and *Strongyloidesmyopotami* and *Trichostrongylusduretteae* Rossin et al., 2006 in the small intestine.

Rossin et al. (2010b) redescribed the metacestode form of *Taeniatalicei* obtained from the peritoneal cavity of two tuco-tuco species including the Southern tuco-tuco, *Ctenomysaustralis* Rusconi, 1934, and the Talas tuco-tuco, *Ctenomystalarum*, from Necochea, Paraje Las Grutas, Buenos Aires Province in Argentina.

From Bolivia, Drabik and Gardner (2019) described *Ancylostomactenomyos* Drabik & Gardner, 2019 from the small intestine of the Bolivian tuco-tuco, *Ctenomysboliviensis* collected from two localities in the Department of Santa Cruz, 3.5 km west of Estación el Pailón and 2 km SSE of Santa Rosa de la Roca, and from Steinbach’s tuco-tuco, *Ctenomyssteinbachi* Thomas, 1907 collected from 2 km S. of Caranda by road in the Department of Santa Cruz.

Gardner et al. (2021) mentioned discovery of a new species of *Mathevotaenia* from the Highland tuco-tuco, *Ctenomysopimus*, collected in 1986 from Huancaroma, Department of Oruro, Bolivia (Gardner et al. 2023). Also from Bolivia, Gardner et al. (2021) also reported *Paraspidodera* nematodes including individuals from the cecae of Anderson’s tuco-tuco, *Ctenomysandersoni*Gardner et al., 2014, the Bolivian tuco-tuco or Cajuchi, *Ctenomysboliviensis* Waterhouse, 1848, Conover’s tuco-tuco, *Ctenomysconoveri* Osgood, 1946, Erica’s tuco-tuco, *Ctenomyserikacuellarae*Gardner et al., 2014, the little Andean forest tuco-tuco, *Ctenomysfrater* Thomas, 1902, Lessa’s tuco-tuco, *Ctenomyslessai*Gardner et al., 2014, Lewis’s tuco-tuco, *Ctenomyslewisi*, Steinbach’s tuco-tuco, *Ctenomyssteinbachi*, and Natterer’s tuco-tuco, *Ctenomysnattereri* Wagner, 1848. In addition, an undescribed species of *Raillietina* was found in the small intestine of *C.erikacuellarae* collected on the experiment station grounds near Monteagudo, Bolivia and *Pudica* sp. Travassos & Darriba, 1929 was also reported from the White-toothed tuco-tuco, *Ctenomysleucodon* Waterhouse, 1848.

The present study reports that during a biodiversity survey in Bolivia in 1986, *Pudicapujoli* Durette-Desset & Tcheprakoff, 1990 was found in a single specimen of the White-toothed tuco-tuco, *Ctenomysleucodon* Waterhouse, 1848, collected from near San Andreas de Machaca, Bolivia.
